# Impact of a virtual antenatal intervention for improved diet and iron intake in Kapilvastu district, Nepal - the VALID randomized controlled trial

**DOI:** 10.3389/fnut.2024.1464967

**Published:** 2024-11-07

**Authors:** Naomi M. Saville, Sanju Bhattarai, Santosh Giri, Suprich Sapkota, Joanna Morrison, Bibhu Thapaliya, Basudev Bhattarai, Samata Yadav, Abriti Arjyal, Andrew Copas, Hassan Haghparast-Bidgoli, Helen Harris-Fry, Reecha Piya, Sushil C. Baral, Sara L. Hillman

**Affiliations:** ^1^Institute for Global Health, University College London (UCL), London, United Kingdom; ^2^HERD International, Lalitpur, Nepal; ^3^London School of Hygiene & Tropical Medicine (LSHTM), London, United Kingdom; ^4^Health Research and Social Development Forum (HERD), Lalitpur, Nepal; ^5^Institute for Women’s Health, University College London (UCL), London, United Kingdom

**Keywords:** anemia, pregnancy, antenatal, virtual counseling intervention, iron intake, diet, Nepal, iron and folic acid

## Abstract

**Introduction:**

Counseling, together with iron and folic acid supplements, can improve hemoglobin levels in pregnant women, but few interventions have tested a virtual method of delivering counseling. We hypothesized that a virtual counseling intervention delivered via a mobile device (mHealth) would prevent and treat anemia, compared with routine antenatal care (ANC).

**Methods:**

Virtual antenatal intervention for improved diet and iron intake (VALID) was a non-blinded parallel group two-arm, individually randomized superiority trial (1:1 allocation). Participants were pregnant women who were married, aged 13–49 years, able to answer questions, 12–28 weeks’ gestation and living in Kapilvastu district, Nepal. Women were randomized to receive routine ANC (control arm), or ANC plus a virtual antenatal intervention of two problem-solving counseling sessions via video call. The primary outcome was iron folic acid (IFA) tablet compliance (consumption on 12 or more days of the previous 14 days). Secondary outcomes were dietary diversity, promoted food consumption, iron bioavailability enhancement, and knowledge of iron-rich foods. Primary logistic regression analysis was by intention-to-treat, adjusting for baseline values.

**Results:**

We enrolled 319 pregnant women (161 control, 158 intervention) from 23 January 2022 to 6 May 2022 and analyzed outcomes in 144 control and 127 intervention women. Compliance with IFA increased in both arms. In the intervention arm, compliance increased by 29.7 percentage points (pp) (49.0–78.7%) and 19.8 pp. in the control arm (53.8–73.6%). Despite the more significant increase in the intervention arm, we found no intervention effect upon IFA compliance (adjusted odds ratio [aOR] 1.33; 95% confidence interval [CI]: 0.75, 2.35; *p* = 0.334), dietary diversity, or ANC visits. The intervention increased knowledge of iron-rich foods (coefficient 0.96; 95% CI: 0.50, 1.41; *p* < 0.001), consumption of promoted foods (aOR: 1.81; 95% CI: 1.08, 3.02; *p* = 0.023), behavior to enhance iron bioavailability (aOR: 4.41; 95% CI: 1.23, 15.83; *p* = 0.023), and coronavirus disease 2019 (COVID-19) knowledge (aOR: 4.06; 95% CI: 1.56, 10.54; *p* = 0.004). The total intervention cost was US$35,193, and the cost per pregnant woman receiving two virtual counseling sessions was US$277.

**Conclusion:**

Virtual counseling can improve antenatal health behaviors, such as the consumption of promoted foods and methods to enhance bioavailability. Improved IFA consumption and ANC attendance may require additional family/community support.

**Clinical trial registration:**

https://www.isrctn.com/ISRCTN17842200, identifier ISRCTN17842200.

## Introduction

Anemia in pregnancy is an intractable problem worldwide (1–3). Severe anemia causes more maternal deaths in South Asia than in any other region (4) and it is doubtful that South Asian countries will meet global targets to reduce anemia by 50% by 2025 (5). In Nepal in 2016, 46% of pregnant women were anemic (hemoglobin [Hb] concentration < 110 g/L) (6), almost unchanged from the 2011 estimate of 48% (7).

Women are recommended to take iron and folic acid (IFA) supplements during pregnancy to reduce the risk of anemia, iron deficiency, and low birth weight (8) and improve child survival (9). However, despite Nepal implementing a routine IFA supplementation program from the second trimester of pregnancy, anemia levels remain alarmingly high, particularly in the Terai (plains) region (6) where thinness and poor diet adversely affect women’s health (10, 11).

Behavior change interventions can play a role in promoting access and adherence to IFA supplementation and improving other determinants of anemia. In Nepal, gender-inequitable intrahousehold dynamics affect access to IFA (12), iron-rich food (10) and ANC (13), suggesting the importance of addressing the family context in reducing anemia (14). Patrilineal marriage practices often result in women moving to the home of their husbands after marriage, where they are lowest in the family hierarchy. They are usually expected to cook for the extended family under the supervision of their mother-in-law, who controls what is cooked and the quantity of food cooked. Cultural norms around maintaining ritual purity of food and the low position of daughters-in-law in the household mean that the cook (usually the daughter-in-law) serves the food and then eats after others have eaten. Women of childbearing age are often expected to be satisfied with eating less and are considered to have lower nutritional needs compared with the men of the family, who tend to work outside the household (15, 16).

Compared with IFA alone, an intervention combining IFA with one short counseling session and provision of an information brochure on anemia was associated with higher hemoglobin concentrations in pregnant women (17). Another nutritional counseling intervention with dietary assessment and menu planning increased hemoglobin concentrations slightly more than general education about hygiene and sanitation, rest/exercise, and danger signs in pregnancy (18).

Significant barriers limit access to ANC and behavior change interventions, such as travel costs and human resource constraints (13). mHealth interventions, defined as *the use of mobile phones and other wireless technology in medical care*, may help to overcome some of these barriers by providing services virtually, notably as mobile phone ownership and network coverage have expanded rapidly across the globe. The potential of mHealth interventions became increasingly apparent during the COVID-19 pandemic, which made providing ANC and face-to-face engagement with health workers difficult. In Nepal, repeated lockdowns, fear of visiting health facilities, and discrimination against frontline workers (19) posed barriers to women’s access to ANC and IFA (20).

Virtually delivered mHealth interventions have so far shown mixed effects on antenatal health behaviors, perhaps because they have had limited participatory elements. We identified nine virtual mHealth interventions aiming to improve food and/or nutrient intakes in pregnancy in low- or middle-income countries (LMICs) from two systematic reviews (21, 22) and our systematic search update (summary in Supplementary Annex 1). All used didactic, information-based models and thus were not tailored to women’s contexts, and most were asynchronously delivered with automated content, which limited their interactivity with participants. Seven of the nine interventions involved reminders to take supplements or attend ANC via SMS, voice messages, or phone calls (23–29); the other two provided educational material via SMS (30) or a smartphone application (31). Impacts were varied, with some showing improvements in adherence to iron tablets (23, 26), ANC (24, 26), diets (30, 31), and Hb (26, 29), while others showed no impact on iron adherence (25, 27, 29) and Hb concentration (23, 25, 28).

The evidence suggests that the virtual mHealth interventions show promise to improve IFA compliance, dietary intakes in pregnancy, and ANC access. However, the interventions tested so far have not been interactive or tailored to women’s contexts and have not included participatory design elements, which we know can have significant impacts on intervention effectiveness and equity of coverage (32, 33) and therefore could improve mHealth effectiveness.

Research is needed to develop and test innovative methods to increase IFA intake and improve dietary quality in Nepal, particularly among hard-to-reach groups. Therefore, we did the “virtual antenatal intervention for improved diet and iron intake” (VALID) individually randomized controlled trial testing the effectiveness of an mHealth dialogical “virtual counseling” intervention delivered via video calls with pregnant women and their families living in Kapilvastu district, Lumbini Province in the Terai. We hypothesized that providing counseling twice in mid-pregnancy would increase compliance to IFA tablets, compared with ANC alone. Although initiating IFA during preconception or in early pregnancy may be beneficial (34), mid-pregnancy was selected as a pragmatic intervention point in this study for three reasons. First, women in Nepal tend only to reveal their pregnancies after the first trimester is complete. Second, compliance with IFA is more challenging to attain in early pregnancy due to nausea during this period. Third, at the time of designing the intervention, IFA was usually only provided free of cost by government health facilities after 16–20 weeks’ gestation to achieve a minimum of 180 days of consumption over the pregnancy.

The primary objective was to assess whether the intervention, in addition to usual government services, increased women’s compliance to IFA supplementation compared with access only to usual government services. As a secondary objective, we assessed impacts on dietary knowledge and practices, access to ANC, and knowledge of COVID-19. We conducted a concurrent process evaluation to understand the intervention’s effects.

## Methods

### Trial design

The trial was a non-blinded parallel group two-arm individually randomized superiority trial with 1:1 allocation. In the control arm, women had access to routine ANC. There were no essential changes to methods after trial commencement or from the published protocol (35).

### Setting

The trial was implemented in 54 rural population clusters of southern Kapilvastu district in the Nepal Terai, bordering the state of Uttar Pradesh in India. Anemia prevalence is high (45%), and IFA in pregnancy is suboptimal: 43% took at least the recommended dose of 180 IFA tablets, 33% took 60–179 tablets, and 24% took <60 tablets (3). IFA is supplied free of charge at primary health services and outreach clinics. Discrimination against young married women is common in the plains of Nepal, and harmful gender norms and intrahousehold hierarchies are significant drivers of women’s poor health and nutrition in this context (10, 12, 14, 15, 36). In Nepal overall, in 2022, 80% of women and 92% of men owned any mobile phone, with 60 and 74%, respectively, owning a smartphone. In rural Lumbini province, 52% of women and 70% of men owned a smartphone, so mobile phone access is high (37). However, since only 61% of rural women in Lumbini province can read a whole sentence (37), their capacity to use smartphones may be limited.

The trial protocol is available as a published paper (35).

### Trial registration

Trial registry name: ISRCTN; registration number: ISRCTN 17842200; registration date: 13 January 2022. URL: https://doi.org/10.1186/ISRCTN17842200.

ISRCTN collects all items from the World Health Organization Trial Registration Data Set.

### Participants

Pregnant women were eligible for the baseline survey if they were aged 13–49 years, could respond to questions, and resided in a study cluster. Additional inclusion criteria for enrolment in the trial included the following: 12–28 weeks’ gestation (estimated from the recall of the last menstrual period or expected date of delivery given by a health worker), no plan to leave the country, and no other pregnant woman in her household already enrolled in the trial. Interviewers identified pregnant women with help from Female Community Health Volunteers, confirmed eligibility, and took written consent.

### Randomization and masking

Health Research and Social Development Forum (HERD) Data Manager implemented stratified block randomization using the “blockrand” package in “R” with four strata defined by the combinations of (i) any IFA consumed in the last 14 days at baseline and (ii) first pregnancy or not. Allocations were sealed into sequentially numbered opaque envelopes, transported to the field office, stored securely, and opened by the monitoring and evaluation manager when interviewers called to ask a participant’s allocation at the end of the baseline interview. After the interviewers had assigned each respondent to a study arm, they visited each pregnant women in the intervention arm to distribute a mobile device. Blinding of trial staff and participants was impossible as they were aware of who was receiving virtual counselling. The details of data management are provided in the trial protocol (35).

### Procedures

After enrolment, researchers delivered a mobile device with a sim card and a data package worth 399 Nepalese rupees (~USD 3) to intervention arm participants. They trained them to use the device. Enrolled women were assigned to one of ten trained counselors who were auxiliary nurse midwives or graduates with >4 years of community-based health intervention experience. Counselors telephoned women to schedule sessions via Zoom at 12–28 weeks’ gestation, and a second session ≥2 weeks later.

The intervention encouraged women and their families to take action to improve dietary practices, IFA compliance, and access to ANC. Using stories and dialogue to trigger reflection and action, counselors supported them in thinking critically about the causes of anemia in pregnancy in their household and community. Stories directly addressed issues identified from formative research (14). After the first session, participants made action plans to address relevant issues for their families. In the second session, action plans were reviewed/discussed, and a second plan was made. At the end of each session, we sent the action plan and some standardized “take-home” messages about dietary guidance, ANC and IFA uptake, and COVID-19 symptoms and prevention to the device via WhatsApp. After the second counseling session, researchers collected the mobile devices. Participants were free to seek concomitant care during pregnancy, irrespective of the trial allocation.

Researchers measured participants’ recall of outcomes and pregnancy symptoms/problems at enrolment (12–28 weeks’ gestation) and endline (at least 49 days later). At baseline, they collected women’s age, gravida, medical history, date of the last menstrual period, and other socioeconomic and demographic data. The data collection tools were programmed onto Android mobile devices in Nepali and English using the CommCare electronic data collection platform. These had in-built jump sequences and value limits to prevent data entry outside plausible ranges. Interviewers followed standard procedures for 24 h dietary recall measurements, and IFA recalls to increase accuracy and minimize the interobserver difference.

### Outcomes

The primary outcome was consumption of IFA on ≥12 of the preceding 14 days. Secondary outcomes were dietary diversity score, consumption of promoted foods, practice of iron bioavailability enhancement, knowledge of iron-rich foods, and number of ANC visits. Exploratory outcomes included understanding why blood tests were needed, knowledge of COVID-19, and timing of ANC. Outcomes were generally derived from multiple questions to generate a count or a binary indicator (Table 1).

**Table 1:** Virtual antenatal intervention for improved diet and iron intake (VALID) trial outcome measures.

VALID trial outcomes	Recorded also at baseline^b^	Recall period	Definition	Variable type	Effect measure to compare arms
Primary outcome					
Compliance with recommended iron and folic acid tablet (IFA) intake	Yes	14 days	IFA consumed on 12 or more days out of the previous 14 days (i.e., on at least 80% of days)	Binary	Odds ratio and difference in proportion^a^
Secondary outcomes					
Dietary diversity	Yes	24 h	Count of the number of food groups consumed in the previous 24 h preceding the endline interview, assessed using the list-based method, out of 10 food groups	Count (0–10)	Difference in mean
Consumption of intervention-promoted foods	Yes	24 h	Any consumption of green leafy vegetables, meat, or fish	Binary	Odds ratio and difference in proportion^a^
Practicing one or more methods to enhance bioavailability	Yes	7 days	Recalled one or more of the following: using lemon or other vitamin C-rich foods with meals, eating sprouted grains or pulses, avoiding tea/coffee 1 h on either side of meals, or spreading meat-eating over two eating occasions rather than one	Binary	Odds ratio and difference in proportion^a^
Knowledge of iron-rich foods	No	N/A	Count of iron-rich food groups correctly recalled	Count (0–9)	Difference in mean
ANC visits	No	Baseline to endline	Antenatal care (ANC) visits between enrolment and endline interview	Count (0–4)	Difference in mean
Exploratory outcomes					
Understanding of why blood tests are taken at antenatal check-ups.	Yes	N/A	The proportion of those women who had a blood test at ANC who could correctly explain one or more reasons for having a blood test	Binary	Odds ratio and difference in proportion^a^
Knowledge of COVID-19 symptoms, precautions, and vulnerability	No	N/A	The proportion of women who could correctly identify at least one vulnerable group, at least three coronavirus disease 2019 (COVID-19) control/prevention measures, and at least three COVID-19 symptoms	Binary	Odds ratio and difference in proportion^a^
ANC visits at the right time for her gestational age	Yes	Pregnancy to date	Whether the woman had her ANC visits at 2, 4, 6, and 8 months or not	Binary	% by arm (no effect measure)

a Formal testing is linked to the odds ratio, but the effect is also expressed as a difference in proportion.

b Items recorded at baseline are adjusted for in analyses.

Counselors and interviewers recorded adverse events and informed field managers. COVID-19 infections were tracked among participants by prospectively filling in COVID-19 symptoms forms before interactions but were not reported as adverse events.

### Statistical analysis

Our target sample size was 150 participants in each arm (300). We undertook power calculations for two scenarios, where the control arm prevalence of the primary outcome (consuming IFA on at least 12 of the preceding 14 days) was either (a) 67% or (b) 50%. Assuming a 10% loss to follow-up in each arm, the sample size gives 80% power to detect a 15 percentage points increase in the primary outcome in scenario (a) and a 16.7 pp. increase in scenario (b).

Our primary analysis was by intention-to-treat (i.e., “as randomized”) following a prespecified Statistical Analysis Plan approved by the Trial Steering Committee (Supplementary Annex 2). All confidence intervals were 95% and two-sided. Statistical tests were two-tailed at the 5% significance level. Analyses of the primary outcome and other binary outcomes were based on logistic regression, leading to adjusted odds ratios (aORs) and 95% CI. We also expressed the intervention effect as an adjusted difference with 95% CI based on marginalization, that is, the difference in predicted outcome prevalence from our regression model between allocating all participants to intervention and all to control. We used linear regression with robust standard errors for count outcomes.

To report data completeness, we considered the primary outcome to be missing where the woman was unavailable, moved away, or withdrew consent but to be not applicable if she had a miscarriage, abortion, or stillbirth. We do not impute any missing values of the primary outcome, as the strongest predictor of the outcome is IFA compliance at baseline, which we have included as a covariate in our regression models.

The analysis of the primary outcome (IFA consumed on ≥12 out of the previous 14 days) was adjusted for the same outcome as reported at baseline and gravida (0, 1+) because these are the design factors that together defined the randomization strata. Another “fully adjusted analysis” adjusted for further factors that we expected to affect the outcome are education, age of the pregnant woman, and gestational age at the endline interview. This was considered a secondary analysis because, despite potentially addressing any residual imbalance between arms, adjustment for many factors may reduce precision. Analysis of secondary outcomes followed the same approach. Both the primary and fully adjusted analyses additionally adjusted for the baseline value of the outcome (if measured at baseline). For count outcomes, the baseline values (where available) were adjusted as a linear term.

A second analyst checked the code used to derive the primary outcome and all adjustment factors in the primary analysis. Preliminary analysis of distribution shape in a dataset without trial arm informed choice of regression method for count outcomes. We examined covariate standard errors to check for collinearity between covariates and model stability. Analyses were conducted using Stata version 17.

We undertook subgroup analyses of the primary outcome, presenting effect measures in women who had and had not, consumed IFA on ≥12 of the previous 14 days at baseline. We also tested the interaction between this factor and the arm. No interim analyses were planned or conducted.

We collected process evaluation data using mixed methods to describe the delivery of the intervention and fidelity to plans, to understand the context and mechanisms of how the intervention worked or failed. The detailed process evaluation results are presented elsewhere (38).

We estimated the costs of designing and implementing the intervention from a program provider perspective. We collected cost data from HERD international accounts, staff time use surveys, and interviews. The time horizon for the analysis was 14 months, which was divided into 7 months of startup and 5 months of implementation. All costs were presented in 2022 US$, using the exchange rates of 131.5 for Nepal and 1.2 for the United Kingdom. Detailed cost analysis will be published separately.

### Patient and public involvement statement

Our study was designed during the acute COVID-19 pandemic, so lockdowns and restrictions on community engagement activities limited our capacity to engage patients and the public in the design of the intervention. However, the virtual counseling intervention was adapted from that designed for a previous trial in the same study population which was stalled due to the pandemic (35). In this study, we undertook extensive public involvement through meetings with municipality leaders and collected extensive formative qualitative data from potential participants in the trial, which informed the content and design of the counseling (12, 14).

## Results

**Figure 1:**
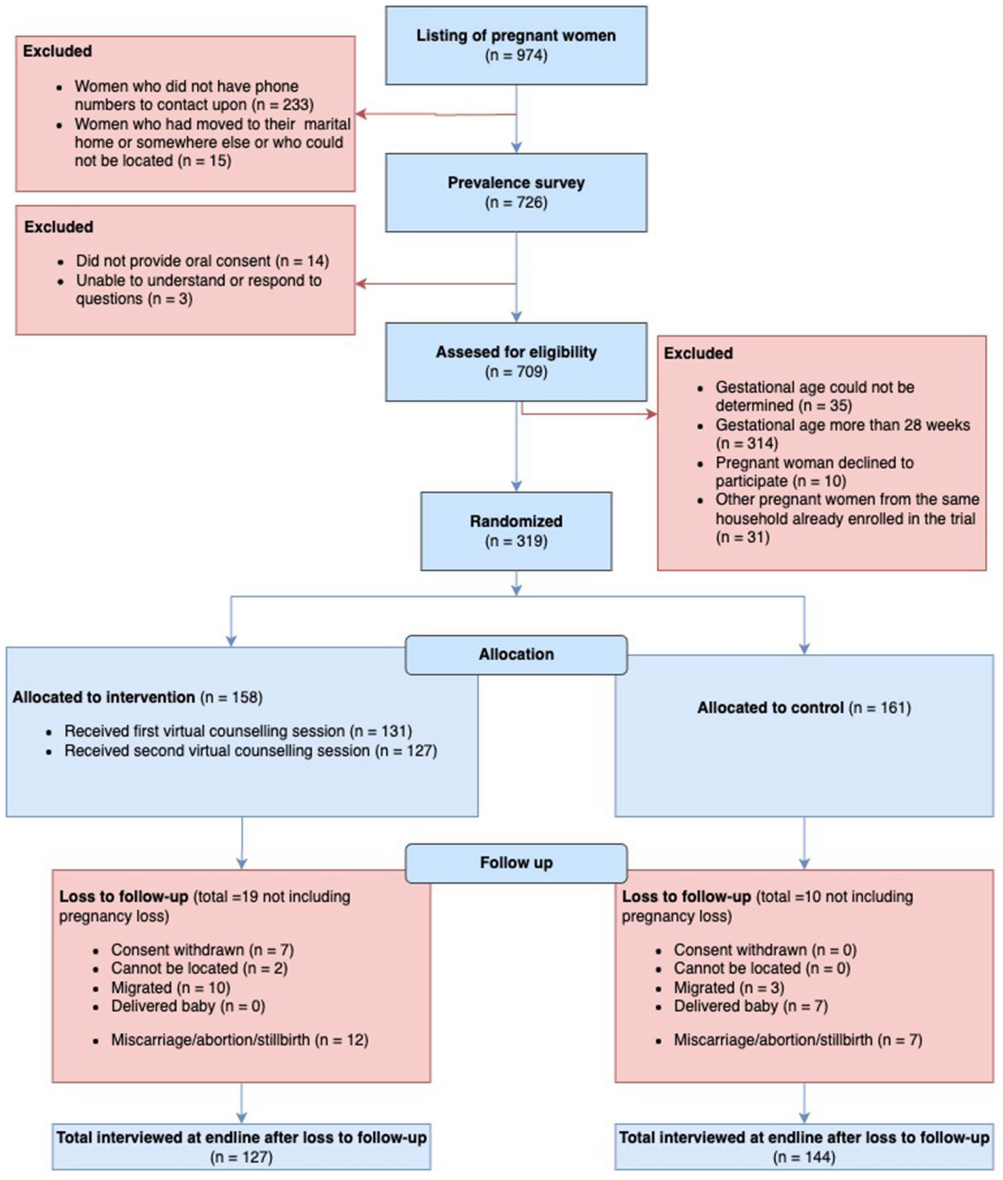
The consolidated standards of reporting trials (CONSORT) flowchart of trial recruitment and retention.

Participant flow (Consolidated Standards of Reporting Trials [CONSORT] diagram) is illustrated in Figure 1. Enrolment ran from 14 January 2022 to 23 February 2022. The counseling sessions ran from 23 January 2022 to 6 May 2022, and the follow-up ranged from 9 March 2022 to 7 June 2022. The trial ended when all women who consented and could be located had been followed up. 319 pregnant women enrolled, 161 in control, and 158 in intervention arms. We obtained IFA compliance for 158 and 155 women at baseline and for 144 control and 127 intervention women at endline, 89.4 and 80.4% of enrolled, respectively.

Miscarriage or abortion amounted to 7 pregnancies in control and 12 in intervention. No stillbirths were recorded. Excluding pregnancy losses, loss to follow-up was 10 (6.2%) in control and 19 (12.0%) in intervention arms. Reasons for losses are detailed in the participant flow diagram but were mostly due to migration, delivering the baby, and, in the case of intervention, withdrawal of consent. No maternal deaths were reported.

Characteristics of those lost to follow-up (or who experienced pregnancy loss) versus those retained by the study arm are provided in Supplementary Annex 3. More multiparous and more illiterate women were lost in the intervention arm than in the control arm.

Baseline characteristics are summarized by the arm in Table 2 and were mostly well balanced between arms. Participants were mainly of Madheshi ethnicity from “Middle Madheshi,” Muslim or Dalit groups. Around two-thirds of the household heads were farmers with landholdings <2 hectares, whereas one in five households was landless. Half (51%) of women could read, and less than one quarter were educated to secondary level or above. Over half of the respondents had smartphone access, but 28% had no phone access, and others used push-button phones. Gestational age at enrolment was a median of 18.4 weeks (IQR: 14.9, 22.6) in the control and 17.5 weeks (IQR: 14.1, 23.4) in the intervention arm. The baseline–endline gap was a median of 55 (IQR: 51, 61) days and did not differ by arm.

**Table 2:** Characteristics of women enrolled in the trial by arm.

Characteristic	Control			Intervention			Total		
	*N*	Frequency	%	*N*	Frequency	%	*N*	Frequency	%
Caste									
Dalit	161	31	19.3	158	24	15.2	319	55	17.2
Janajati		4	2.5		1	0.6		5	1.6
Muslim		44	27.3		48	30.4		92	28.8
Middle Madheshi		78	48.4		81	51.3		159	49.8
Brahmin/Chettri		4	2.5		4	2.5		8	2.5
Primary source of household income									
Farming	161	101	62.7	158	108	68.4	319	209	65.5
Animal husbandry		3	1.9		2	1.3		5	1.6
Skilled labor		11	6.8		13	8.2		24	7.5
Unskilled labor		28	17.4		16	10.1		44	13.8
Job/business/other		7	4.3		10	6.3		17	5.3
Remittances		11	6.8		9	5.7		20	6.3
Gravida									
Primigravida	161	35	21.7	158	34	21.5	319	69	21.6
1 previous pregnancy		44	27.3		44	27.8		88	27.6
2 previous pregnancies		39	24.2		24	15.2		63	19.7
3+ previous pregnancies		43	26.7		56	35.4		99	31.0
Pregnant woman’s education									
No schooling	161	51	31.7	158	61	38.6	319	112	35.1
Primary to grade 8		69	42.9		61	38.6		130	40.8
Secondary grade 9 and above		41	25.5		36	22.8		77	24.1
Pregnant woman’s literacy									
Cannot read (ref)	161	71	44.1	158	84	53.2	319	155	48.6
Reads with difficulty or easily		90	55.9		74	46.8		164	51.4
Pregnant woman’s access to a phone									
No phone access	161	49	30.4	158	42	26.6	319	91	28.5
Owns or accesses a push-button phone	161	63	39.1	158	64	40.5	319	127	39.8
Owns or accesses a smartphone	161	90	55.9	158	83	52.5	319	173	54.2
Woman’s knowledge of using the internet									
No knowledge	161	70	43.5	158	66	41.8	319	136	42.6
Some/little experience		66	41.0		72	45.6		138	43.3
Experienced		25	15.5		20	12.7		45	14.1
Continuous variables	*N*	Mean	SD	*N*	Mean	SD	*N*	Mean	SD
Household size	161	8.8	4.9	158	8.8	5.1	319	8.8	5.0
Total number of children (alive)	161	1.5	1.3	158	1.6	1.6	319	1.6	1.5
Age of pregnant woman	161	25.2	4.0	158	25.7	4.8	319	25.5	4.4
Age at marriage	161	18.4	2.4	158	18.3	2.4	319	18.3	2.4
Age at first pregnancy	161	20.4	2.1	158	20.3	2.7	319	20.4	2.4
Age of first menstruation	161	13.5	1.3	158	13.6	1.3	319	13.6	1.3
Continuous variables	*N*	Median	IQR	*N*	Median	IQR	*N*	Median	IQR
Gestational age at enrolment (baseline)	161	18.4	14.9, 22.6	156	17.5	14.1, 23.4	317	18.0	14.4, 22.9
Gestational age at follow-up (endline)	157	26.1	23.1, 30.4	124	26.1	23.3, 30.8	281	26.1	23.1, 30.7

IQR, interquartile range; SD, standard deviation.

Implementation went as planned and followed prespecified protocols (35, 38). A total of 131 women received the first counseling session, and 126 received the second. The median gap between the two sessions was 18 days (IQR: 16, 21). Women were median 21.3 (IQR: 17.3, 24.7) and 23.8 (IQR 20.2, 27.4) weeks’ gestation at first and second counseling, respectively.

Outcomes at baseline and endline are described in Table 3, and Table 4 gives minimally adjusted (primary analysis) regression results. Fully adjusted regression results are shown in Table 5. In the text, we provide ORs and coefficients from the primary analyses.

**Table 3:** Outcomes at baseline and endline.

	Baseline						Endline					
Outcomes	Control			Intervention			Control			Intervention		
Primary outcome	*N*	Frequency	%	*N*	Frequency	%	*N*	Frequency	%	*N*	Frequency	%
Compliance with recommended iron and folic acid tablet (IFA) intake on 12+ out of 14 days	158	85	53.8%	155	76	49.0%	144	106	73.6%	127	100	78.7%
Secondary outcomes	*N*	Mean	SD	*N*	Mean	SD	*N*	Mean	SD	*N*	Mean	SD
Dietary diversity of pregnant women in last 24 h (10 groups)	158	5	1.7	154	4.7	1.7	144	4.7	1.5	127	4.9	1.5
Knowledge of iron-rich foods (maximum 9 foods)	0	na		0	na		144	2.4	1.8	127	3.4	1.9
ANC visits between baseline and endline	0	na		0	na		144	1.2	0.7	127	1.2	0.7
Binary secondary outcomes	*N*	Frequency	%	*N*	Frequency	%	*N*	Frequency	%	*N*	Frequency	%
Consumption of intervention-promoted foods in the last 24 h	158	121	76.6%	154	106	68.8%	144	79	54.9%	127	85	66.9%
Practicing one or more methods to enhance iron bioavailability in the last 7 days	158	138	87.3%	154	135	87.7%	144	130	90.3%	127	124	97.6%
Exploratory outcomes	*N*	Frequency	%	*N*	Frequency	%	*N*	Frequency	%	*N*	Frequency	%
Knows > = 1 reason for getting a blood test (out of all those who had a blood test)	27	21	78.0%	22	13	59.0%	51	34	67.0%	47	37	79.0%
Reasons cited for blood tests												
To check for anemia	27	20	74.0%	22	13	59.0%	51	32	63.0%	47	34	72.0%
To check for blood-borne diseases (HIV, hepatitis)	27	8	30.0%	22	4	18.0%	51	24	47.0%	47	22	47.0%
To check for syphilis	27	4	15.0%	22	3	14.0%	51	24	47.0%	47	19	40.0%
To check blood sugar levels	27	7	26.0%	22	3	14.0%	51	19	37.0%	47	18	38.0%
To see if the blood of the mother and baby are compatible (rhesus factor)	27	2	7.0%	22	2	9.0%	51	1	2.0%	47	4	9.0%
To conduct thyroid (TSH) tests	27	0	0.0%	22	0	0.0%	51	4	8.0%	47	5	11.0%
Knowledge of COVID-19: 3 symptoms, 3 precautions, and 1 vulnerable group	0	na		0	na		144	6	4.2%	127	19	15.0%
ANC visits at the right time for her gestational age	158	28	17.7%	154	37	24.0%	144	44	30.6%	127	34	26.8%

ANC, antenatal care; IQR, interquartile range; na, not applicable; SD, standard deviation; TSH, thyroid stimulating hormone.

**Table 4:** Intervention effect on a) the primary, secondary and exploratory outcomes and b) the primary outcome by baseline compliance.

(a) Intervention effect on the primary and secondary outcomes	Adjusted for baseline IFA and gravida								
Primary outcome	Odds ratio	Lower 95% CI	Upper 95% CI	*p*	*n*	Marginalized difference in proportion	Lower 95% CI	Upper 95% CI	*n* for marginal effect
Compliance with recommended iron and folic acid tablet (IFA) intake	1.33	0.75	2.35	0.334	271	0.05	−0.05	0.15	299

	Adjusted for baseline outcome value (where available), baseline IFA and gravida								
Secondary outcomes (counts)	Coefficient (difference)	Lower 95% CI	Upper 95% CI	*p*	*n*				
Dietary diversity	0.19	−0.15	0.54	0.272	271				
Knowledge of iron-rich foods	0.96	0.50	1.42	<0.001	271				
ANC visits between baseline and endline	0.02	−0.15	0.20	0.81	271				
Binary secondary outcomes	Odds ratio	Lower 95% CI	Upper 95% CI	*p*	*n*	Marginalized difference in proportion	Lower 95% CI	Upper 95% CI	*n* for marginal effect
Consumption of intervention-promoted foods	1.81	1.08	3.02	0.023	271	0.13	0.02	0.24	298
Practicing one or more methods to enhance iron bioavailability, including avoiding tea close to meals	4.41	1.23	15.83	0.023	271	0.08	0.01	0.16	298
Exploratory outcomes	Odds ratio	Lower 95% CI	Upper 95% CI	*p*	*n*	Marginalized difference in proportion	Lower 95% CI	Upper 95% CI	*n* for marginal effect
Understanding of why blood tests are taken at antenatal check-ups (among those who had blood tests)	1.93	0.27	13.93	0.513	35	0.11	−0.22	0.44	48
Knowledge of COVID-19 symptoms, precautions, and vulnerability	4.06	1.56	10.54	0.004	271	0.11	0.03	0.20	299

(b) Intervention effect on the primary outcome by baseline compliance	Adjusted for gravida									
Disaggregated analyses for women complying and not complying at baseline	Odds ratio	Lower 95% CI	Upper 95% CI	*p*	*n*	Marginalized difference in proportion	Lower 95% CI	Upper 95% CI	*n* for marginal effect	*p*-value for interaction between IFA compliance at baseline and study arm^a^
Women taking IFA on 12/14 days at baseline	0.84	0.37	1.90	0.675	147	−0.03	−0.16	0.10	159	0.14
Women not taking IFA on 12/14 days at baseline	2.04	0.90	4.64	0.088	124	0.14	−0.01	0.29	140	

a Interaction between IFA compliance at baseline and study arm based on the model adjusting for gravida only.

ANC, antenatal care; COVID-19, coronavirus disease 2019; CI, confidence interval; IFA, iron folic acid.

**Table 5:** Fully adjusted analyses of (a) intervention effect on the primary, secondary, and exploratory outcomes, and (b) intervention effect on the primary outcome by baseline compliance.

(a) Intervention effect on outcomes	Adjusted for baseline IFA, gravida, education, age of pregnant woman, gestational age at follow-up								
Primary outcome	Odds ratio	Lower 95% CI	Upper 95% CI	*p*	*n*	Marginalized difference in proportion	Lower 95% CI	Upper 95% CI	*n* for marginal effect
Compliance with recommended iron and folic acid tablet (IFA) intake	1.31	0.73	2.35	0.372	265	0.05	−0.06	0.15	277

	Adjusted for baseline outcome (where available), baseline IFA, gravida, education, age of pregnant woman, gestational age at follow-up								
Secondary outcomes (counts)	Coefficient (difference)	Lower 95% CI	Upper 95% CI	*p*	*n*				
Dietary diversity	0.21	−0.14	0.56	0.24	265				
Knowledge of iron-rich foods	1.06	0.59	1.52	<0.001	265				
ANC visits between baseline and endline	0.04	−0.13	0.21	0.648	265				
Binary secondary outcomes	Odds ratio	Lower 95% CI	Upper 95% CI	*p*	*n*	Marginalized difference in proportion	Lower 95% CI	Upper 95% CI	*n* for marginal effect
Consumption of intervention-promoted foods	1.91	1.13	3.22	0.015	265	0.15	0.03	0.26	277
Practicing one or more method to enhance bioavailability including avoiding tea close to meals	4.59	1.26	16.74	0.021	265	0.09	0.01	0.17	277
Exploratory outcomes	Odds ratio	Lower 95% CI	Upper 95% CI	*p*	*n*	Marginalized difference in proportion	Lower 95% CI	Upper 95% CI	*n* for marginal effect
Understanding of why blood tests are taken at antenatal check-ups (among those who had blood tests)	3.20	0.35	29.21	0.302	35	0.18	−0.13	0.50	48
Knowledge of COVID-19 symptoms, precautions and vulnerability	4.25	1.61	11.27	0.004	265	0.11	0.04	0.19	277

(b) Intervention effect on the primary outcome by baseline compliance	Adjusted for baseline IFA, gravida, education, age of pregnant woman, gestational age at follow-up									
Disaggregated analyses for women complying and not complying at baseline	Odds ratio	Lower 95% CI	Upper 95% CI	*p*	*n*	Marginalized difference in proportion	Lower 95% CI	Upper 95% CI	*n* for marginal effect	*p*-value for interaction between IFA compliance at baseline and study arm^a^
Women taking IFA on 12/14 days at baseline	0.93	0.40	2.18	0.871	144	−0.01	−0.14	0.12	149	0.225
Women not taking IFA on 12/14 days at baseline	1.78	0.77	4.12	0.179	121	0.11	−0.05	0.27	128	

a Interaction between IFA compliance at baseline and study arm based on a model adjusted for gravida, education, age of pregnant woman, and gestational age at enrolment.

ANC, antenatal care; COVID-19, coronavirus disease 2019; CI, confidence interval; IFA, iron folic acid.

**Figure 2:**
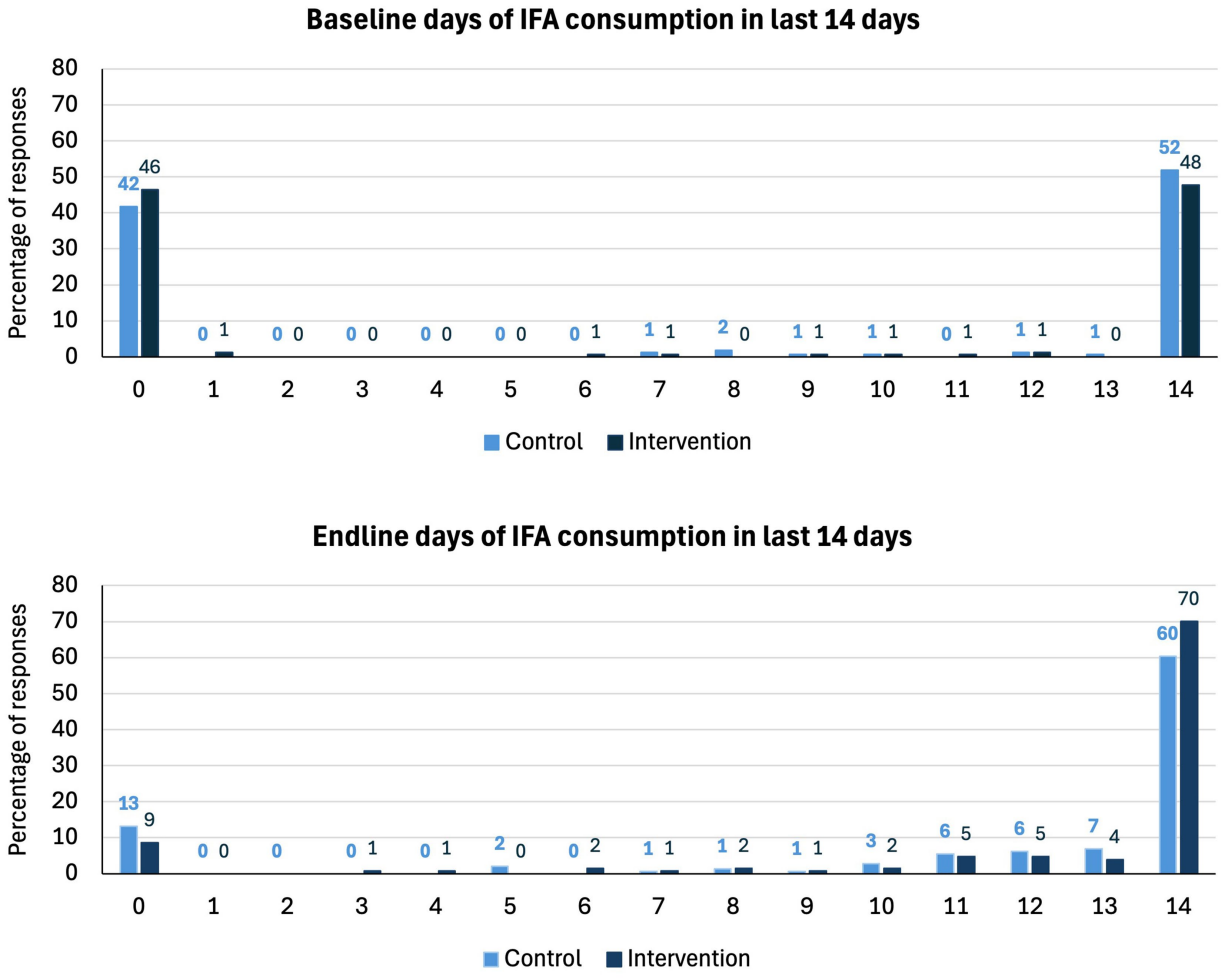
Bar chart of the number of days of IFA consumption in the last 14 days by arm at baseline and endline by trial arm.

There was no effect of the intervention on IFA compliance (odds ration [OR]: 1.33; 95% CI: 0.75, 2.35; *p* = 0.334). Compliance increased from 53.8 to 73.6% (an increase of 19.8 pp) in the control arm and from 49.0 to 78.7% (29.7 pp) in the intervention arm (Table 3). Figure 2 illustrates the number of days of IFA consumption in the last 14 days by the arm at baseline and endline and displays a strong bimodal distribution. At both baseline and endline, women either mostly complied with daily consumption or did not consume IFA at all. At the endline, a small number of women reported missing a few days of consumption, whereas at baseline, almost all women either recalled 0 or 14 days of consumption.

Among secondary outcomes, we found no apparent intervention effect on dietary diversity score or number of ANC visits. We found that the intervention increased dietary knowledge and improved nutritional practices. Women in the intervention arm could name one more iron-rich food on average than those in control (control mean 2.4; intervention mean 3.4 foods; coefficient 0.96; 95% CI: 0.50, 1 0.41; *p* < 0.001). The proportion of women consuming intervention-promoted foods was higher in the intervention than control, aOR: 1.81; 95% CI: 1.08, 3.02; *p* = 0.023. The odds of practicing iron bioavailability enhancement (squeezing lemon on food, avoiding tea wholly or within 1 h of mealtimes, or spreading meat eating out between two eating occasions rather than one) was also higher (aOR: 4.41; 95% CI: 1.23, 15.83; *p* = 0.023).

Among exploratory outcomes, we found that knowledge of COVID-19 increased (aOR: 4.06; 95% CI: 1.56, 10.54; *p* = 0.004). The proportion who could recall three COVID-19 symptoms, three precautions, and one COVID-19 vulnerable group was higher at the endline in the intervention (15%) than in the control participants (4.2%). Understanding of the reasons for blood tests at ANC did not differ. The proportion of women who had their ANC visits at the right time (i.e., at 2, 4, 6, and 8 months) was 30.6% in the control and 26.8% in the intervention arm.

Ancillary analyses are given in Table 5. We did not find a differential effect of the intervention on IFA compliance between women who were or were not complying with IFA supplements at baseline.

Table 6 shows reported reasons why women did not take IFA and women’s recall of action plans. The main reasons were lack of time to visit health facilities and side effects. Approximately 80% of women receiving the counseling session recalled making an action plan. The most common actions were drinking adequate water, going for ANC, and consuming IFA tablets. Half of the women made action plans to improve their consumption of vitamin C and iron-rich foods after the first counseling session. Some planned actions related to pregnancy problems that were not dietary (e.g., back pain and leg cramps).

**Table 6:** Reasons for not taking iron and recall of action plans cited by pregnant women.

Reasons for not taking IFA cited by the woman	Control baseline *n* = 66		Control endline *n* = 22		Intervention baseline *n* = 71		Intervention endline *n* = 13	
	Frequency	%	Frequency	%	Frequency	%	Frequency	%
Didn’t have time to go to health facility	14	21	11	50	10	14	5	38
Last time I took iron tablets in pregnancy I got side effects	2	3	6	27	3	4	4	31
The woman ran out of iron tablets	0	0	1	5	1	1	1	8
Doctor advised not to take IFA	0	0	0	0	0	0	1	8
There was no problem so did not see the need for IFA	0	0	0	0	0	0	1	8
Went to parental home	0	0	0	0	0	0	1	8
Did not take IFA due to Ramadan	0	0	1	5	0	0	1	8
Planning to take later in pregnancy but it is not time to take them yet	45	68	1	5	51	72	0	0
Health post ran out of IFA/did not provide IFA, or health worker unavailable	3	5	2	9	2	3	0	0
Not interested in taking iron tablets	0	0	0	0	2	3	0	0
Didn’t know the importance of (IFA)	0	0	1	5	0	0	0	0

Recall of action plans by women in the intervention arm who could remember making an action plan	Recall of first virtual counseling *n* = 102		Recall of second virtual counseling *n* = 96	
Following advice related to dietary and/or IFA intake:	Frequency	%	Frequency	%
Drinking adequate water	86	84	81	84
Getting ANC check-up	80	78	79	82
Regular consumption of IFA tablets	71	70	76	79
Importance of Vitamin C and consumption of iron-rich foods	54	53	69	72
Dealing with weakness during pregnancy	23	23	25	26
Dealing with gastritis (indigestion) during pregnancy	25	25	23	24
Dealing with loss of appetite during pregnancy	10	10	12	13
What to do when I dislike certain foods	9	9	6	6
Reducing or stopping tea drinking	13	13	5	5
Eat larger servings and more often	0	0	2	2
Taking lemon	2	2	1	1
Soak pulses (Bengal gram)	1	1	1	1
Others (eat beetroot/pickles with meals/take sag and dairy)	0	0	3	3
Take calcium supplements/deworming	0	0	2	2
Dealing with piles	1	1	0	0
Actions related to other problems experienced by pregnant women	Frequency	%	Frequency	%
Dealing with back pain during pregnancy	13	13	14	15
Leg cramps/pain during pregnancy	6	6	10	10
Avoid lifting/carrying heavy loads	9	9	4	4
Get an ultrasound	0	0	3	3

ANC, antenatal care; COVID-19, coronavirus disease 2019; IFA, iron folic acid.

A virtual intervention may be more cost-effective than a face-to-face intervention because of the time and travel costs saved. We could not undertake a cost-effectiveness analysis because of the lack of significant difference in compliance between arms, but the calculated total cost of the intervention was US$35,193, and cost efficiency or costs per pregnant woman receiving two virtual counseling sessions was US$277. However, at scale and under routine, non-research condition costs would potentially be lower.

## Discussion

The VALID mHealth virtual counseling intervention did not improve IFA compliance, dietary diversity, or number of ANC visits relative to the control. However, women’s knowledge and consumption of intervention-promoted iron-rich foods and behaviors to enhance iron bioavailability were significantly higher in the intervention arm compared to the control. Although the increase in the proportion complying with 12 or more days of consumption out of 14 was higher in the intervention than in the control arm, the odds of compliance did not differ between arms. Given the intervention did not increase IFA compliance, ANC, or dietary diversity, it is unlikely that the changes in consumption of iron-rich and iron-bioavailability-enhancing foods alone would have led to improved anemia levels for pregnant women in the intervention arm.

The cost of the intervention was US$277 per participant covered. However, if scaled up, costs would be lower due to economies of scale. To our knowledge, there is no published cost data on community-based implementations of mHealth virtual antenatal counseling tools in LMICs. In addition, comparing the costs of this virtual antenatal counseling intervention with other mHealth counseling tools is challenging because of differences in the type of intervention (e.g., virtual counseling meetings or SMS and call reminders), scale, and cost approach. Research from high-income settings suggests that satisfaction levels are similar between face-to-face and virtual counseling, but face-to-face counseling is usually of a longer duration (39). Given the modest effects seen, it is questionable whether it would be justified to undertake a repeat trial of the same intervention with a larger sample size powered to detect a smaller difference between arms.

Although the intervention did not improve IFA compliance, compliance improved in both arms over time. For those who did not take IFA, a primary reason cited by participants in both study arms was not having time to go to the health facility to get IFA tablets. Convenient access, family support to accompany pregnant women to ANC, and the ability to take time out from their responsibilities are necessary to enable women to get IFA tablets (38). Formative data showed a lack of trust in government health services by husbands in particular (12). This may have restricted women’s access to IFA since women’s intrahousehold status is often low, and they may not have been able to negotiate with the family to visit the health facility. Women living far from a health facility, those from poor or marginalized families, or those living in conservative families may benefit from virtual counseling, especially if supplemented with targeted support from Female Community Health Volunteers (FCHVs). FCHVs could help to identify particularly vulnerable women in prepregnancy and then make home visits to deliver IFA which may improve compliance (40).

Despite attempting to engage family members in the intervention to accompany women to ANC, improve their access to iron-rich foods, and comply with IFA, this was difficult because of the different working patterns of men and women and the small screen limitations. This lack of family engagement may have affected women’s access to IFA, thereby contributing to a lack of intervention impact on compliance. Hence, the future interventions could benefit from providing additional behavior change content that can be accessed at a time that is convenient for the whole family (e.g., videos, group chats, or SMS content), and/or by engaging with communities to address harmful gender norms (41, 42), and engaging with families face-to-face to problem-solve (38). Our approach might have been enhanced by a concomitant provision of asynchronously delivered content (43), and/or by community-focused interventions such as participatory learning and action (PLA) groups. PLA groups have been successful at increasing dietary diversity for pregnant women and children in rural India (44, 45) and for pregnant women in Nepal (46). Unfortunately, this was not feasible in the context of COVID-19. Health systems strengthening to improve the supply and distribution of IFA and quality of ANC, plus community engagement may help to increase trust in ANC and create an enabling environment to address anemia, especially if harmful gender and social norms can be tackled at the community level (12, 14, 38, 47).

In contrast to Galloway’s review, which found that 10% of women reported that they stopped taking IFA due to side effects (48), at the endline, we found that ~3% of respondents (and 27–31% of non-compliant women) cited side effects as their reason for not taking IFA. Although counselors were trained on strategies to manage side effects, this could receive more focus in future interventions. There is potential to include targeted advice about addressing side-effects of IFA in Ministry of Health revisions to the ANC guidance.

Our results also showed conflicting impacts on diets. While dietary diversity did not change, consumption of iron-rich food (meat, fish, or green leafy vegetables) increased among women in the intervention arm compared to the control. The lack of effect on overall dietary diversity may be explained by the substitution of foods, for instance, if meat is substituted for other vegetable-based foods. Additionally, households may have “channeled” meat to pregnant women, as was found in a trial of PLA women’s groups in Nepal, which demonstrated increased odds of pregnant women consuming more animal–source foods than their household head in intervention relative to control areas (46).

Women were also more likely to enact behaviors to increase the bioavailability of iron in food in the intervention arm. Process evaluation data showed that the advice not to drink tea within 1 h of meals and to combine green leaves and pulses with sources of vitamin C (e.g., lemon) during meals was novel and interesting for women and that these actions were within their control (38). Similarly, knowledge of COVID-19 symptoms, precautions, and vulnerability, was significantly higher at endline in intervention (15%) than in control (4.2%). This may also be because the information was new and relevant. Innovative, practical, and low-cost strategies for pregnant women and their families are necessary to maintain family and community focus on preventing anemia.

Women appeared to be less able to change ANC (and IFA) access which depended on others’ support. Some advice, which was sent via WhatsApp messages, was well received. This suggests that the platform of intervention delivery should be one that is most familiar and easy to use for women and their families. mHealth messaging to remind women about dietary and supplement intake during their pregnancy (30, 31, 49) combined with participatory problem-solving with families and communities might enable women to access ANC and IFA.

### Meaning of the study: possible explanations and implications for clinicians and policymakers

Our trial of an mHealth virtual counseling intervention showed limited benefits to IFA compliance and dietary intake in pregnancy in a region of Nepal with a high burden of anemia where women have low literacy levels and limited access to mobile devices. Unreliable/inaccessible mobile phone networks and lack of confidence in the use of mobile devices impeded the implementation of our intervention, and some women did not have the agency to move to areas with better reception or to speak freely in front of family members (38). Moreover, engaging family members was difficult because they were often away from home during the day, and/or engaging via the small screen of the tablet was challenging (38). Our findings are generalizable to other areas of South Asia where women may have access to smartphones but have limited skills in how to use them and low levels of agency. The intervention might have more application in contexts where there is better mobile coverage, women routinely use smartphones for video calls, and women’s families are enabled to support them.

Our mHealth virtual counseling intervention did not improve IFA compliance, dietary diversity, or ANC access but did improve pregnant women’s dietary knowledge and some dietary practices. This may be due to a lack of access to IFA, the need for family and community support, and the side effects of IFA.

### Strengths and weaknesses of the study

Our study implemented a highly contextualized intervention, which was informed by detailed, mixed-methods research with potential participants, and used a rigorous randomized controlled trial design to evaluate impact. We achieved high fidelity in implementation despite the logistical challenges of scheduling and delivering a synchronously delivered, novel mHealth virtual counseling approach and achieved satisfactory levels of follow-up despite COVID-19 restrictions still being in place during implementation. We implemented a detailed and rigorous process evaluation to understand the effect of our intervention which has been reported elsewhere (38). Our study is among the first to report the costs of an mHealth virtual counseling intervention in LMICs.

Our study has some limitations. Due to the government IFA provision beginning in the fourth month of pregnancy, we were not able to begin our intervention in early or prepregnancy despite this being a potentially good approach. Our outcomes were all self-reported, which is subject to respondent bias. For example, most women recalled taking IFA daily or not at all, but this could be subject to bias in recall. We could not measure hemoglobin due to COVID-19 physical contact restrictions. Due to COVID-19, our timeline for intervention was limited. The short time gap between counseling sessions and the primary outcome measurement meant the intervention had limited time to affect behaviors. Given that IFA compliance over a 14-day period was >70% in both study arms at the endline future studies should be powered for modest impacts and could target starting IFA earlier in pregnancy.

Dialogical counseling encouraged women to strategize with the counselors, but engaging family members proved difficult (38). Counselors had to make multiple phone calls to pregnant women to arrange virtual counseling sessions (38) which increased the cost and decreased the feasibility of the intervention. Some women required multiple visits from researchers to learn to use the mobile device, which made the intervention logistically challenging (38). Frustrations with these intervention processes may have led to a higher loss of follow-up in the intervention group than in the control.

### Unanswered questions and future research

Given the modest effects seen, future studies should test integrated complex interventions at the health system, community, family, and individual levels to promote problem-solving and develop enabling environments to address maternal anemia. Health system strengthening could improve IFA supply and distribution, and enhanced community engagement and gender transformative interventions are required to address community barriers to ANC and IFA compliance. Given the relatively high short-term compliance in both intervention and control arms, future work could investigate whether compliance is sustained throughout pregnancy and, if not, how to maintain it. The intervention was done mid-pregnancy. It is conceivable that beginning counseling periconceptually or during early pregnancy might enhance the effect. Since the anemia burden persists in Nepal despite relatively high IFA compliance, future studies need to identify other potential biological drivers of anemia in this setting.

## Conclusion

The VALID trial is the first randomized controlled trial to test the impact of two dialogical counseling sessions delivered via video call on a mobile device loaned to pregnant women in the rural plains of Nepal. The intervention improved knowledge and consumption of promoted iron-rich foods, adoption of dietary behaviors to increase iron bioavailability, and knowledge of COVID-19 symptoms, precautions, and vulnerability. There was no significant increase in compliance with IFA, dietary diversity, or the number of ANC visits between baseline and endline in the intervention, compared with the control arm, which received routine ANC.

Our research shows that virtual counseling with women alone can improve some health behaviors but is not sufficient to bring changes in behaviors that are contextually driven and often rely on family support in this context. Virtual (video call) counseling informed by formative research is a useful supplement to ANC to improve knowledge among pregnant women about how to increase the bioavailability of iron and iron-rich foods and could be included in multicomponent interventions to address anemia in pregnancy.

Future interventions to address drivers of anemia in pregnancy could supplement virtual counseling with asynchronously delivered mHealth approaches, home visits among hard-to-reach groups, and gender-transformative community-focused interventions.

## Data Availability

The datasets presented in this study can be found in the UCL research data repository. Deidentified individual participant data that underlie the findings in this article (and data dictionaries) can be found here: https://doi.org/10.5522/04/27077962.v1.
