# Supplementary material for: Impact of a virtual antenatal intervention for improved diet and iron intake in Kapilvastu district, Nepal - the VALID randomized controlled trial
Source: Front Nutr. 2024 Nov 7;11:1464967. doi: 10.3389/fnut.2024.1464967 (PMC11580260; doi:10.3389/fnut.2024.1464967)
Supplement: Supplementary file 1 [file Table_1.DOCX]

**Supplementary Annex 1. Summary of literature review search strategy and papers found**

One systematic review showed that ANC uptake and/or iron supplementation may improve with Short-Message Service reminders, but many studies were of poor quality (1).

We identified a systematic review of mHealth interventions targeting pregnancy intakes in low- and lower middle-income countries (LLMICs) including 4 articles with 2 that evaluated change in adherence to iron supplementation as the main outcome (2). Short-messaging service (SMS) and phone call reminders to pregnant women to take supplements and attend antenatal care (ANC), showed improved iron intake in two studies, but no impact on Haemoglobin (Hb) levels.

We updated this review from 1 January 2018 to 11 March 2023 using identical search terms: mHealth interventions; pregnancy; iron and folic acid (IFA) supplementation and dietary intake; in low and lower middle-income countries and added search terms “randomi*ed control trial” or “randomi*ed contro*ed trial” or “RCT”. We searched MEDLINE, EMBASE, CINAHL, Cochrane, Web of Science, Scopus, Global Index Medicus and Maternity and Infant Care. The initial search returned 248 articles. We excluded study protocols, studies that did not include pregnant women, were not situated in low- or lower middle-income countries (LLMICs), did not test a mHealth intervention, or did not report on nutrition behaviours as outcomes (e.g., dietary intake, nutritional supplements intake, eating habits).

Five eligible studies were identified including 4 RCTs (3 individual, 1 cluster) and 1 quasi-RCT (cluster). The interventions tested included SMS messaging, mobile phone call reminders, web- and smartphone-based applications. In Iran, SMS reminders to prediabetic pregnant women were associated with improved food group intake (fruits, vegetables, meats, dairy products) and reduced oil and sweets intake (3). Mobile phone calls to remind anaemic pregnant women in India to take iron tablets resulted in increased Hb levels from baseline to endline and a non-significant increase in iron tablet compliance (4). In Indonesia a web-based application with information on pregnancy and nutrition, brainstorming with peers and staff, and videos of peer models describing their experiences of pregnancy and childbirth was used during four face-to-face sessions and women were expected to use it at home. This did not significantly improve IFA compliance compared to face-to-face antenatal education (5). A smart-phone application for Accredited Social Health Activists (ASHAs) in India to use during face-to-face home visits was associated with a significant improvement in consumption of the prescribed dose of IFA (6). Another smart-phone app targeting middle-class pregnant women in Pune, India provided information in the form of videos, tips, and quizzes and increased dietary diversity and knowledge on anaemia (7).

1. Lee SH, Nurmatov UB, Nwaru BI, Mukherjee M, Grant L, Pagliari C. Effectiveness of mHealth interventions for maternal, newborn and child health in low- and middle-income countries: Systematic review and meta-analysis. J Glob Health. 2016;6(1):010401.

2. Saronga NJ, Burrows T, Collins CE, Ashman AM, Rollo ME. mHealth interventions targeting pregnancy intakes in low and lower-middle income countries: Systematic review. Matern Child Nutr. 2019;15(2):e12777.

3. Abbaspoor Z, Amani A, Afshari P, Jafarirad S. The effect of education through mobile phone short message service on promoting self-care in pre-diabetic pregnant women: A randomized controlled trial. Journal of Telemedicine & Telecare. 2020;26(4):200-6.

4. Sontakke P, Dwidmuthe KS, Kawathalkar A, Bhalerao A. Effect of Mobile Phone Call Reminders With Standard Therapy Versus Standard Therapy Alone on Compliance With Iron Supplementation in Antenatal Women With Iron Deficiency Anemia: A Randomized Controlled Trial. Cureus Journal of Medical Science. 2022;14(9).

5. Ariyani NW, Wirawan IMA, Pinatih GNI, Kusuma A. The effect of an application-based educational intervention with a social cognitive theory model on pregnant women in Denpasar, Bali, Indonesia: a randomized controlled trial. OSONG PUBLIC HEALTH AND RESEARCH PERSPECTIVES. 2022;13(2):153-61.

6. Choudhury A, Choudhury M. Mobile for Mothers mHealth Intervention to Augment Maternal Health Awareness and Behavior of Pregnant Women in Tribal Societies: Randomized Quasi-Controlled Study. JMIR mHealth and uHealth. 2022;10(9):e38368.

7. Dieteren C, Sarkar S, Saharan S, Bonfrer I. Effects of a smartphone application on maternal health knowledge and dietary diversity among pregnant women in India: a randomized single center pilot study. Journal of Global Health Reports. 2022;6.
