# Supplementary material for: Impact of a virtual antenatal intervention for improved diet and iron intake in Kapilvastu district, Nepal - the VALID randomized controlled trial
Source: Front Nutr. 2024 Nov 7;11:1464967. doi: 10.3389/fnut.2024.1464967 (PMC11580260; doi:10.3389/fnut.2024.1464967)
Supplement: Supplementary file 2 [file Table_2.DOCX]

**Supplementary Annex 2. Statistical Analysis plan**

**A randomised controlled trial of a Virtual Antenatal Intervention for improved Diet and Iron intake in Kapilbastu district, Nepal: VALID**

**Protocol version**: 1.0, 04 Apr 2022

**Trial registration**: ISRCTN 17842200; Date of registration: 13 Jan 2022.

URL: <https://doi.org/10.1186/ISRCTN17842200>

**Statistical Analysis Plan (SAP)**

**Version 1.0, 27.05.2022**

| **Authorised by:** | **Signature** | **Date** |
| --- | --- | --- |
|  |  |  |
|  |  |  |
| Chief investigator  Nepal Co-Principal Investigators 1 and 2  Trial Statistician |  | 27 May 2022  27 May 2022  27 May 2022 |

**Table of Contents**

[1. ABBREVIATIONS 4](#_Toc104536376)

[1. Background and design 4](#_Toc104536377)

[1.1. Scope of this SAP 4](#_Toc104536378)

[1.2. Aim and objectives 4](#_Toc104536379)

[1.3. Population studied, eligibility criteria 5](#_Toc104536380)

[1.4. Interventions and trial design 5](#_Toc104536381)

[1.5. Consent for participation and enrolment in the trial 6](#_Toc104536382)

[1.6. Randomisation 6](#_Toc104536383)

[1.7. Blinding: 6](#_Toc104536384)

[2. OUTCOME MEASURES 6](#_Toc104536385)

[2.1. Adverse events 8](#_Toc104536386)

[3. DATA 8](#_Toc104536387)

[3.1. Data collection 8](#_Toc104536388)

[3.2. Data storage and management 8](#_Toc104536389)

[3.3. Analysis dataset 8](#_Toc104536390)

[3.4. Data verification 9](#_Toc104536391)

[3.5. Data coding 9](#_Toc104536392)

[3.6. Derivation and coding of primary and secondary outcomes 9](#_Toc104536393)

[Primary outcome 9](#_Toc104536394)

[Secondary and exploratory outcomes 9](#_Toc104536395)

[3.7. Derivation and coding of exposure and adjustment factors 9](#_Toc104536396)

[4. Sample size calculation 9](#_Toc104536397)

[5. Analysis Principles 10](#_Toc104536398)

[5.1. Intention-to-treat (ITT) or per-protocol? 10](#_Toc104536399)

[5.2. Confidence Intervals and *p*-values 10](#_Toc104536400)

[5.3. Baseline comparability 10](#_Toc104536401)

[5.4. Adjustment for design and contextual factors 10](#_Toc104536402)

[5.5. Losses to follow-up and missing data 10](#_Toc104536403)

[5.6. Regression models and effect measures 10](#_Toc104536404)

[5.7. Subgroup and dose-response analyses 11](#_Toc104536405)

[5.8. Descriptive summaries 11](#_Toc104536406)

[6. Analysis and reporting details 11](#_Toc104536407)

[6.1. Recruitment, intervention uptake and follow-up 11](#_Toc104536408)

[6.2. Analysis software 11](#_Toc104536409)

[6.3. Checking 11](#_Toc104536410)

[6.4. Regression diagnostics 12](#_Toc104536411)

[7. Interim analyses 12](#_Toc104536412)

[7.1. Regular reports to TSC-DMC 12](#_Toc104536413)

[8. Tables and Figures for the primary publication 12](#_Toc104536414)

[8.1. Tables 12](#_Toc104536415)

[8.2. Graphs 18](#_Toc104536416)

[9. References 19](#_Toc104536417)

# 1. ABBREVIATIONS

| **Abbreviation/Acronym** | **Meaning** |
| --- | --- |
| ANC | Antenatal care |
| CAPPT | Comprehensive Anaemia Programme and Personalized Therapies |
| DMC | Data Monitoring Committee |
| FCHV | Female Community Health Volunteer |
| HERD | Health Research and Social Development Forum International |
| ID | (Trial) Identifier |
| IFA | Iron folic acid |
| LMP | Last Menstrual Period |
| NEC | Nutrition education and counselling |
| PLA | Participatory Learning and Action |
| PW | Pregnant woman |
| QR | Quick response |
| SAP | Statistical analysis plan |
| SOP | Standard operating procedure |
| TSC | Trial Steering Committee |
| UCL | University College London |
| WHO | World Health Organisation |

# Background and design

Despite evidence that iron and folic acid (IFA) supplements can improve anaemia in pregnant women, uptake in Nepal is suboptimal. We hypothesised that providing virtual antenatal counselling twice in mid-pregnancy, would increase compliance to IFA tablets in Kapilbastu, Nepal during the COVID-19 pandemic compared with ANC alone.

## Scope of this SAP

This SAP specifies the study results to be included in the **primary results publication**, and analyses required for monitoring the safety of the trial, but does not cover the health economics or process/implementation analyses described in the overall study Protocol.

## Aim and objectives

In this trial we test whether a virtual counselling intervention offered to pregnant women living in Kapilbastu district in the plains of Lumbini province, Nepal could offer a feasible, affordable, scalable, and equitable solution to increase consumption of iron-folic acid supplements.

The primary objective of the Virtual Antenatal Intervention for Improved Diet and iron intake (VALID) trial is to assess whether providing pregnant women with an antenatal virtual counselling intervention, in addition to usual government services, increases women’s compliance to IFA supplementation, compared with pregnant women who only had access to usual government services. Compliance is defined as: IFA consumed on 12 out of the last 14 days before the endline interview (which is 49-70 days after baseline). We hypothesised that the antenatal virtual counselling intervention would increase pregnant women’s compliance to IFA tablets.

Secondary objectives are as follows:

To assess whether providing an antenatal virtual counselling intervention to pregnant women improves

- Dietary diversity,
- Timing and number of antenatal visits (ANC),
- Consumption of recommended iron-rich foods
- Practice of iron absorption-enhancing behaviours
- Knowledge of iron-rich foods and anaemia risks in pregnancy.
- Knowledge of COVID symptoms, prevention, and vulnerable population groups

Definitions regarding interpretation of these objectives into specific outcome measures are given later in the SAP.

## Population studied, eligibility criteria

The trial is being implemented in 54 clusters in Kapilbastu district, Lumbini Province of Nepal, in the Western Terai (plains) of Nepal, bordering Uttar Pradesh state of India. The district population is 569,844 and literacy rates of women and men are 45% and 65% respectively ^1^. Hinduism is the main religion, followed by Islam and the predominant ethnicity is Madhesi, many of whom speak Awadhi language. Anaemia prevalence is high (45%) and IFA in pregnancy is sub-optimal: 43% took at least the recommended dose of 180 IFA tablets, 33% took 60-179 tablets, and 24% took <60 tablets ^2^.

Study clusters are rural areas of southern Kapilbastu district that do not adjoin the main East-West highway that traverses Nepal; with no major market; 1100 -3199 projected population; surrounded with a buffer zone of non-study clusters; and >50% Madhesi (plains ethnicity) as per the pre-trial census.

Pregnant women are eligible for a baseline survey if they are aged 13-49 years, able to respond to questions, reside in a study cluster, and consent to participate. Additional inclusion criteria for enrolment in the trial include: 12 to 28 weeks’ gestation (estimated from recall of last menstrual period or expected date of delivery given by a health worker), does not plan to leave the country during the 5 weeks since enrolment, and no other pregnant woman in her household already enrolled in the trial.

## Interventions and trial design

The trial is a non-blinded parallel group two-arm individually randomised superiority trial with 1:1 allocation. In the control arm women have access to routine ANC.

After enrolment, interviewers call pregnant women in the intervention arm to arrange to deliver a tablet to them. Each tablet holds a sim card and has a data package which is topped up as necessary. Enrolled women are assigned to one of ten counsellors, who are recruited and trained auxiliary nurse midwives. Counsellors call the women to schedule their tailored virtual antenatal counselling sessions via Zoom. Pregnant women are offered their first counselling session after enrolment, at 12 to 28 weeks’ gestation, and the second counselling session is around two weeks later, at 14 to 31 weeks’ gestation.

The counselling aims to support women and their families to take actions to change dietary practices, take IFA and deworming tablets, and access ANC. Counselling is conducted with pregnant women and their families to support them to think critically about the causes of anaemia in pregnancy in their household and community, using stories and inductive questioning to trigger dialogue and reflection ^3^. Stories directly address issues identified from formative research ^4^. At each session, pregnant women (and their families if they attend) are engaged in a cycle of action and reflection whereby they discuss common issues that affect women in pregnancy and examples of actions for improving intake of IFA and deworming tablets, accessing ANC, and dietary practices. At the end of the first counselling session, the pregnant women and families make specific action plans to address the issues that are relevant for their family. In the second counselling session these action plans are reviewed and further discussed to support women and their families to address the issues and a second action plan is made. The tablets are collected back by interviewers after completing two counselling sessions.

Participants are free to seek concomitant care during pregnancy irrespective of their trial allocation.

## Consent for participation and enrolment in the trial

To enrol participants, interviewers identify pregnant women in their study areas with help from FCHVs. They confirm eligibility, take written consent, and then phone the office to receive the random allocation.

## Randomisation

A stratified block randomisation process is used to allocate pregnant women enrolled into the trial intervention or control arm. To balance the trial arms by two strong pre-identified determinants of IFA consumption, gravida and baseline IFA consumption status, randomisation is done within each of four strata: (IFA yes/1^st^ pregnancy); (IFA Yes/ not 1^st^ pregnancy); (IFA no/1^st^ pregnancy) and (IFA no/ not 1^st^ pregnancy). For each stratum a separate allocation sequence is prepared. To make the sequence unpredictable, random permutation of the allocations within blocks is conducted and block sizes vary randomly between sizes 8, 6 and 4 using “blockrand” package in R programming software. Then, sequential allocations are sealed into sequentially numbered opaque envelopes by the project coordinator in Kathmandu, transported to the field office, stored in a locked cabinet, and opened by the monitoring and evaluation manager when the enumerators call to find out a pregnant woman’s allocation at the end of the baseline interview.

## Blinding:

After assignment of pregnant women to study arms, blinding of trial staff and participants is impossible, since interviewers distribute of tablet devices to pregnant women in the intervention arm. In most cases the same interviewers collect baseline and endline data for women in both trial arms.

# OUTCOME MEASURES

The primary outcome of the trial is consumption of IFA on at least 80% of the previous 2 weeks (i.e., on >=12 out of 14 days recalled). Other outcomes are given in Table 1.

**Table 1. VALID outcome measures**

| **VALID TRIAL OUTCOMES** | **Recorded also at baseline**^#^ | **Recall period** | **Definition** | **Variable type** | **Effect measure to compare arms** |
| --- | --- | --- | --- | --- | --- |
| **PRIMARY OUTCOME** |  |  |  |  |  |
| Compliance with recommended iron and folic acid tablet (IFA) intake | Yes | 14 days | IFA consumed on 12 or more days out of the previous 14 days (i.e., on at least 80% of days) | Binary | Odds ratio and Difference in Proportion* |
| **SECONDARY OUTCOMES** |  |  |  |  |  |
| Dietary diversity | Yes | 24 hours | Count of the number of food groups consumed in the previous 24 hours preceding the endline interview, assessed using the list-based method, out of 10 food groups | Count (0 to 10 ) | Difference in Mean |
| Consumption of intervention-promoted foods | Yes | 24 hours | Any consumption of green leafy vegetables, meat or fish | Binary | Odds ratio and Difference in Proportion* |
| Practicing one or more to enhance bioavailability | Yes | 7 days | Recalled one or more of the following: using lemon or other vitamin C-rich foods with meals, eating sprouted grains or pulses, avoiding tea/coffee 1 hr either side of meals, or spreading meat-eating over two eating occasions rather than one. | Binary | Odds ratio and Difference in Proportion* |
| Knowledge of iron-rich foods | No | N/A | Count of iron-rich food groups correctly recalled | Count (0 to 9) | Difference in Mean |
| ANC visits | No | Baseline to endline | Antenatal (ANC) visits between enrolment and endline interview | Count (0 to 4) | Difference in Mean |
| **EXPLORATORY OUCOMES** |  |  |  |  |  |
| Understanding of why blood tests are taken at antenatal check-ups. | Yes | N/A | Proportion of those women who had a blood test at ANC who could correctly explain one or more reason for having a blood test. | Binary | Odds ratio and Difference in Proportion* |
| Knowledge of COVID-19 symptoms, precautions and vulnerability | No | N/A | Proportion of women who could correctly identify at least one vulnerable group, at least 3 COVID control/prevention measures and at least 3 COVID symptoms | Binary | Odds ratio and Difference in Proportion* |
| ANC visits at the right time for her gestational age | Yes | Pregnancy to date | Whether the woman had her ANC visits at 2, 4, 6, and 8 months or not | Binary | % by arm (no effect measure) |

* Formal testing is linked to the odds ratio, but the effect is also expressed as a difference

^#^ Items recorded at baseline are adjusted for in analyses

## Adverse events

Counsellors and interviewers record adverse events (including maternal deaths or COVID-19 cases) and inform field managers who submit an adverse events form to the PIs.

# DATA

## Data collection

Interviewers measure outcomes on trial participants at enrolment (12 to 28 weeks’ gestation) and endline (ideally 49 to 70 days after baseline, otherwise up to delivery). All outcomes are recalled by trial participants during interviews and recorded in electronic questionnaires. Additionally, at baseline we collect the woman’s age, gravida, medical history, date of the last menstrual period, pregnancy symptoms/ problems, and other key socioeconomic and demographic information.

The data collection tools are programmed onto mobile devices in Nepali and English on Android operating system tablets or mobile phones using the CommCare electronic data collection platform. These have in-built jump-sequences and value limits to prevent entry of data outside plausible ranges.

Interviewers follow standard procedures for 24-hour dietary recall measurements and IFA recall to ensure that measurements are accurate and inter-observer difference minimised.

The monitoring team meets monthly and Kathmandu-based team members provide support as needed. Interviewers log any problems with electronic forms and the data team make corrections reversibly in the data using Stata data cleaning ‘do’ files as required.

## Data storage and management

We use CommCare’s case management system that enables interviewers and counsellors to follow up pregnant women for data collection and intervention and promotes participant retention. Interviewers and FCHVs hold paper lists of potential pregnant women prior to enrolment. Data collected on electronic forms are locally stored before they are synchronised (encrypted) to the CommCare cloud server.

The data manager in Kathmandu downloads the data daily from the CommCare cloud server onto their server, imports the data into Stata and runs ‘do’ files for data pseudonymising, labelling and recoding. The data are stored on password-protected, encrypted, secure server computers in lockable rooms at the HERD International Kathmandu office. We back up data from physical servers onto secure cloud servers daily, and onto external hardware-encrypted hard drives each week. Person-identifiable data are stored in separate encrypted files and are only used to generate follow-up lists for authorised field team members. All other data are pseudonymised.  Study arm is encoded but not labelled. After data collection is completed, we will delete VALID data from laptops, tablets, and the CommCare server.

Data cleaning is completed by identifying outliers and removing as required. HERD International follows international and NHRC policies for archiving of data in Nepal. We will make the pseudonymised dataset open access by using the UCL data sharing platform as per MRC guidelines. Any request for archived person-identifiable data will go through the trial management team in alignment with provisions in the consent forms.

## Analysis dataset

Database lock will only occur once the last participant’s data has been collected, data cleaning has been completed and all data queries are closed. A frozen dataset will be created by the study Data Manager for statistical analysis.

## Data verification

Basic data checks are performed by the HERD International Data Management team trial Data Manager periodically during the trial. Additional range, consistency and missing data checks will be performed when the datasets for analysis are constructed, as appropriate, before the statistical analysis is performed. All variables will be examined for unusual, outlying, unlabelled or inconsistent values.

Any problems with study data will be queried with HERD International Data Management Team. If possible, data queries will be resolved; although it is accepted that due to administrative reasons and data availability a small number of problems will continue to exist. These will be minimised.

## Data coding

Details of the variables, including variable coding lists are included in the metadata related to the CommCare database.

## Derivation and coding of primary and secondary outcomes

### Primary outcome

The primary outcome is a direct measurement of IFA tablets consumed recoded into a binary indicator from one question, with no further coding involved.

To demonstrate how the primary outcome is derived we will graphically present the number of days of IFA consumption in last 14 days by arm (bar chart) at baseline and follow-up by trial arm

### Secondary and exploratory outcomes

These are generally derived from multiple questions, but the derivation is simple in each case, either a count or a binary indicator that one or more items have been reported.

## Derivation and coding of exposure and adjustment factors

The primary exposure, allocation to intervention, is a direct part of the randomisation. Note that gravida and baseline consumption of IFA are the basis of the stratified randomisation. For each outcome the baseline value of the outcome will also be adjusted for.

**Table 2. Derivation of exposure and adjustment factors**

| **Confounders / covariates to adjust for in analyses:** | **How derived for analysis** |
| --- | --- |
| Literacy of pregnant woman | Binary indicator of able to read with ease or difficulty or not, derived from asking the respondent to read a sentence |
| Gravida (primigravida or not) | Direct question, grouped 0, 1+ for analysis |
| Age of pregnant woman at enrolment in years | Not derived, used as a linear term |
| Gestational age at follow-up measurement in weeks | Not derived, used as a linear term |
| Baseline consumption of IFA for 12+ of the 14 days preceding baseline | Binary measure |

# Sample size calculation

Our target sample size is 300 pregnant women, 150 in each arm. With at least 270 followed up (90% follow-up rate in each arm), we have 80% power to detect a 15 percentage-point increase in IFA compliance (the proportion of women consuming IFA on at least 12 out 14 days preceding the endline survey) if the control arm compliance prevalence is 66-68% (or 16.7 percentage point difference if control arm compliance is only 50%).

# Analysis Principles

## Intention-to-treat (ITT) or per-protocol?

Our primary analysis is by intention-to-treat, according to randomisation. We planned to conduct a per-protocol analysis but almost all women in the intervention arm (that completed a follow-up questionnaire) received both the intended virtual counselling sessions so this is not possible. For the same reason the initially planned ‘dose-response’ subgroup analysis is not possible (see later).

## Confidence Intervals and *p*-values

All confidence intervals will be 95% and two-sided. Statistical tests will be two-tailed and applied at the 5% significance level. The significance level for secondary outcomes will not be adjusted for multiple testing.

## Baseline comparability

‘Baseline’ characteristics (i.e., characteristics of women at enrolment) will be summarised by trial arm (Table 3).

## Adjustment for design and contextual factors

The main analysis of the primary outcome (IFA consumed on 12 or more days out of the previous 14 days) will be adjusted for the same outcome as reported at baseline and gravida (0, 1+) which together define the strata used in randomisation. As a secondary analysis of this outcome we will also present a ‘fully adjusted analysis’ adjusted in addition for further factors expected to predict the outcome: education, age, and gestational age at follow-up interview. This fully adjusted analysis is considered secondary because it is thought that adjustment for so many factors many reduce precision (although potentially reducing ‘bias’ from chance imbalance).

Analysis for secondary outcomes follows the same approach, but both the primary and fully adjusted analysis will be adjusted in addition for the baseline value of the secondary outcome (if this was measured at baseline). For count outcomes the baseline values (where available) will be adjusted as a linear term provided the distribution approximates to normal.

At the time of finalising the first draft of the statistical analysis plan we examined the distributions of outcome variables and covariates in an interim dataset without study arm so as to be able to propose choices for the regression method and how covariates are included. However, final decisions as to how exactly baseline information will be used as covariates in regression models for the outcomes (i.e. as linear or not, how categories may be defined) will be taken after data collection is complete, again using a dataset without trial arm to avoid any possibility of introducing bias. A baseline factor may not be adjusted for at all in the event of sparse data, e.g., if fewer than 20 participants in one category of a binary baseline factor, or if within a category of a baseline factor the outcome takes only one value (i.e. all compliant or all non-compliant in IFA use).

## Losses to follow-up and missing data

We expect very low missing data in our baseline indicators, but we anticipate some women will not complete a follow-up interview and hence have missing outcome measures at follow-up. We consider data as missing where the woman is unavailable, has moved away, or withdrew consent but we do not regard data as ‘missing’ if due to miscarriage. We are not planning imputation of the primary or any other outcome since there is little information on which to base the imputation other than baseline value of the outcome, which we are including as a covariate in our regression models.

We will report loss to follow-up by selected key baseline characteristics and outcomes as outlined in the Appendix table, separately by trial arm.

## Regression models and effect measures

Analysis of the primary outcome is based on logistic regression, leading to adjusted odds ratios and 95% CI (generated using the Wald method). We will also express the intervention effect as an adjusted difference with 95% CI based on marginalisation (the Stata margins command, applied to all participants including those with missing endline data, to give the hypothetical effect had all women received the intervention and all not).

Analysis of secondary binary outcomes (Table 1) will follow a similar methodology, with adjusted marginalised differences presented alongside odds ratios for binary outcomes. For count outcomes linear regression will be used leading directly to a mean difference, and marginalisation is not required. For these count outcomes, because the range of potential values is constrained, some of the assumptions of linear regression will inevitably be violated. For this reason we will use robust standard errors for the regression analysis of these outcomes.

## Subgroup and dose-response analyses

We plan subgroup analyses of the primary outcome only, presenting effect measures in women who had, and had not, at baseline consumed IFA on 12 or more of the previous 14 days. We will also test the interaction between this binary factor at baseline and randomisation arm based on a logistic regression model.

We had planned a dose-response analysis for the primary outcome only within the intervention arm, based on whether the women had received 0, 1 or 2 virtual counselling sessions. However, nearly all women received 2 counselling sessions so this analysis will not be possible.

## Descriptive summaries

Descriptive summaries of outcomes at follow-up will also be provided (planned dummy tables are included at the end of this SAP), with effect measures and summary statistics as in Table 1. Baseline characteristics will also be summarised by arm.

# Analysis and reporting details

## Recruitment, intervention uptake and follow-up

We will report the numbers recruited to the trial and followed-up by arm, and the level of intervention exposure experienced in the intervention arm. The timing of the follow-up interview (i.e. gestational age) will be reported for each arm (median and IQR).

A CONSORT style flow chart will be produced, reporting the number of participants by arm with missing data, and the number for whom follow-up was not applicable (e.g., due to miscarriage or termination).

## Analysis software

Analyses will be conducted using Stata V16 or v17.

## Checking

The code used to derive the primary outcome and all adjustment factors in the primary analysis of the primary outcome will be checked by a second analyst.

## Regression diagnostics

We shall check for model stability primarily through examination of standard errors for covariates, with large standard error or convergence problems indicating a problem of model instability. All final decisions as to functional form (transformation, categorisation) for covariates and choice of regression method for count outcomes will be made based on a dataset without trial arm.

# Interim analyses

There are no planned interim analyses of intervention effectiveness.

## Regular reports to TSC-DMC

The trial will recruit and collect data quickly and there are no planned interim reports.

# Tables and Figures for the primary publication

## Tables

Example dummy results tables to be included in the trial paper are given below. Areas shaded grey are to be populated with numbers.

Table 3. (Table 1 in trial paper). Characteristics of women enrolled to the trial by arm

| **Characteristic** | **Control** | | | **Intervention** | | | **Total** | | |
| --- | --- | --- | --- | --- | --- | --- | --- | --- | --- |
|  | N | Freq. | % | N | Freq. | % | N | Freq. | % |
| **Caste** |  |  |  |  |  |  |  |  |  |
| Dalit |  |  |  |  |  |  |  |  |  |
| Janajati |  |  |  |  |  |  |  |  |  |
| Muslim |  |  |  |  |  |  |  |  |  |
| Madheshi (plains groups) |  |  |  |  |  |  |  |  |  |
| Brahmin/Chettri |  |  |  |  |  |  |  |  |  |
| **Income sources** |  |  |  |  |  |  |  |  |  |
| Farming |  |  |  |  |  |  |  |  |  |
| Animal husbandry |  |  |  |  |  |  |  |  |  |
| Skilled labour |  |  |  |  |  |  |  |  |  |
| Unskilled labour |  |  |  |  |  |  |  |  |  |
| Remittances |  |  |  |  |  |  |  |  |  |
| Job / Business /Other |  |  |  |  |  |  |  |  |  |
| **Landholding sizes (adjusted for land value)** |  |  |  |  |  |  |  |  |  |
| Landless |  |  |  |  |  |  |  |  |  |
| Small landholding (< 3 Bighas) |  |  |  |  |  |  |  |  |  |
| Medium (3.1 to 7 bighas) |  |  |  |  |  |  |  |  |  |
| Large (More than 7 bighas) |  |  |  |  |  |  |  |  |  |
| **Livestock ownership** |  |  |  |  |  |  |  |  |  |
| Cows/Buffalo |  |  |  |  |  |  |  |  |  |
| Goats |  |  |  |  |  |  |  |  |  |
| Chickens/Ducks |  |  |  |  |  |  |  |  |  |
| **Previous pregnancies** |  |  |  |  |  |  |  |  |  |
| Primigravida |  |  |  |  |  |  |  |  |  |
| 1 previous pregnancy |  |  |  |  |  |  |  |  |  |
| 2 previous pregnancies |  |  |  |  |  |  |  |  |  |
| 3+ previous pregnancies |  |  |  |  |  |  |  |  |  |
| **Pregnant Woman’s Education** |  |  |  |  |  |  |  |  |  |
| No schooling |  |  |  |  |  |  |  |  |  |
| Primary to grade 8 |  |  |  |  |  |  |  |  |  |
| Secondary grade 9 and above |  |  |  |  |  |  |  |  |  |
| **Pregnant woman’s literacy** |  |  |  |  |  |  |  |  |  |
| Cannot read |  |  |  |  |  |  |  |  |  |
| Reads with difficulty or easily |  |  |  |  |  |  |  |  |  |
| **Husband’s Education** |  |  |  |  |  |  |  |  |  |
| No schooling |  |  |  |  |  |  |  |  |  |
| Primary to grade 8 |  |  |  |  |  |  |  |  |  |
| Secondary grade 9 and above |  |  |  |  |  |  |  |  |  |

| **Characteristic** | **Control** | | | **Intervention** | | | **Total** | | |
| --- | --- | --- | --- | --- | --- | --- | --- | --- | --- |
|  | N | Freq. | % | N | Freq. | % | N | Freq. | % |
| **Woman's access to a phone** |  |  |  |  |  |  |  |  |  |
| No phone access |  |  |  |  |  |  |  |  |  |
| Owns / accesses Push button phone |  |  |  |  |  |  |  |  |  |
| Owns / accesses Smart phone |  |  |  |  |  |  |  |  |  |
| **Woman's knowledge of using a internet on a phone** |  |  |  |  |  |  |  |  |  |
| No knowledge |  |  |  |  |  |  |  |  |  |
| Some / little experience |  |  |  |  |  |  |  |  |  |
| Experienced |  |  |  |  |  |  |  |  |  |
| **Woman has access to the internet (if knows how to use internet)** |  |  |  |  |  |  |  |  |  |
| Not at all |  |  |  |  |  |  |  |  |  |
| Via WIFI |  |  |  |  |  |  |  |  |  |
| Via smart phone |  |  |  |  |  |  |  |  |  |
| **Pregnant woman’s** | N | Mean | SD | N | Mean | SD | N | Mean | SD |
| Household size |  |  |  |  |  |  |  |  |  |
| No of live children |  |  |  |  |  |  |  |  |  |
| Age at enrolment |  |  |  |  |  |  |  |  |  |
| Age at marriage |  |  |  |  |  |  |  |  |  |
| Age at first pregnancy |  |  |  |  |  |  |  |  |  |
| Age at menarche |  |  |  |  |  |  |  |  |  |

Table 4. (Table 2. in trial paper) Outcomes at baseline and endline

|  | BASELINE | | | | | | ENDLINE | | | | | |
| --- | --- | --- | --- | --- | --- | --- | --- | --- | --- | --- | --- | --- |
| **VALID TRIAL OUTCOMES** | Control | | | Intervention | | | Control | | | Intervention | | |
| **PRIMARY OUTCOME** | N | Freq | % | N | Freq | % | N | Freq | % | N | Freq | % |
| Compliance with recommended iron and folic acid tablet (IFA) intake on 12+ out of 14 days |  |  |  |  |  |  |  |  |  |  |  |  |
| **SECONDARY OUTCOMES** | N | Mean | SD | N | Mean | SD | N | Mean | SD | N | Mean | SD |
| Dietary diversity of pregnant woman in last 24 hrs (10 groups) |  |  |  |  |  |  |  |  |  |  |  |  |
| Knowledge of iron-rich foods (max 9 foods) |  |  |  |  |  |  |  |  |  |  |  |  |
| ANC visits between baseline and endline |  |  |  |  |  |  |  |  |  |  |  |  |
| **Binary secondary outcomes** | N | Freq | % | N | Freq | % | N | Freq | % | N | Freq | % |
| Consumption of intervention-promoted foods |  |  |  |  |  |  |  |  |  |  |  |  |
| Practicing one or more to enhance bioavailability |  |  |  |  |  |  |  |  |  |  |  |  |
| **EXPLORATORY OUCOMES** | N | Freq | % | N | Freq | % | N | Freq | % | N | Freq | % |
| Knows reasons for blood test (out of all those who had a blood test) |  |  |  |  |  |  |  |  |  |  |  |  |
| Reasons cited for blood tests |  |  |  |  |  |  |  |  |  |  |  |  |
| To check for anaemia |  |  |  |  |  |  |  |  |  |  |  |  |
| To check for blood-borne diseases (HIV, Hepatitis) |  |  |  |  |  |  |  |  |  |  |  |  |
| To check for syphilis |  |  |  |  |  |  |  |  |  |  |  |  |
| To check blood sugar levels |  |  |  |  |  |  |  |  |  |  |  |  |
| Other (rhesus, thyroid, etc) |  |  |  |  |  |  |  |  |  |  |  |  |
| Knowledge of COVID-19 symptoms, precautions and vulnerability |  |  |  |  |  |  |  |  |  |  |  |  |
| ANC visits at the right time for her gestational age |  |  |  |  |  |  |  |  |  |  |  |  |

Table 5. (Table 3. in trial paper) Intervention effect on the primary and secondary outcomes

| **VALID TRIAL OUTCOMES** | Adjusted for baseline IFA and gravida | | | Adjusted for baseline IFA, gravida, education, age of pregnant woman, gestational age at follow-up | | |
| --- | --- | --- | --- | --- | --- | --- |
| **PRIMARY OUTCOME** | **Odds ratio (95% CI)** | **p value** | **Marginalised difference (95% CI)** | **Odds ratio (95% CI)** | **p value** | **Marginalised difference (95% CI)** |
| Compliance with recommended iron and folic acid tablet (IFA) intake |  |  |  |  |  |  |
|  | Adjusted for baseline outcome value (where available), baseline IFA and gravida | | | Adjusted for baseline outcome (where available), baseline IFA, gravida, education, age of pregnant woman, gestational age at follow-up | | |
| **SECONDARY OUTCOMES (counts)** | **Difference (95% CI)** | **p value** |  | **Difference (95% CI)** | **p value** |  |
| Dietary diversity |  |  |  |  |  |  |
| Knowledge of iron-rich foods |  |  |  |  |  |  |
| ANC visits |  |  |  |  |  |  |
| **Binary secondary outcomes** | **Odds ratio (95% CI)** | **p value** | **Marginalised difference (95% CI)** | **Odds ratio (95% CI)** | **p value** | **Marginalised difference (95% CI)** |
| Consumption of intervention-promoted foods |  |  |  |  |  |  |
| Practicing one or more to enhance bioavailability |  |  |  |  |  |  |
| **EXPLORATORY OUCOMES** | **Odds ratio (95% CI)** | **p value** | **Marginalised difference (95% CI)** | **Odds ratio (95% CI)** | **p value** | **Marginalised difference (95% CI)** |
| Understanding of why blood tests are taken at antenatal check-ups (amongst those who had blood tests) |  |  |  |  |  |  |
| Knowledge of COVID-19 symptoms, precautions and vulnerability |  |  |  |  |  |  |

Table 6. (Table 4. In trial paper) Intervention effect on the primary outcome by subgroup

|  | Adjusted for gravida | | | Adjusted for gravida, education, age of pregnant woman, gestational age at follow-up | | |  |
| --- | --- | --- | --- | --- | --- | --- | --- |
| **PRIMARY OUTCOME** | **Odds ratio (95% CI)** | **p value** | **Marginalised difference (95% CI)** | **Odds ratio (95% CI)** | **p value** | **Marginalised difference (95% CI)** | **p value for interaction between IFA compliance at baseline and study arm*** |
| Women taking IFA on 12/14 days at baseline |  |  |  |  |  |  |  |
| Women not taking IFA on 12/14 days at baseline |  |  |  |  |  |  |  |

* Based on the model adjusting also for gravida only

## Graphs

Figure 1. CONSORT flowchart of trial recruitment and retention


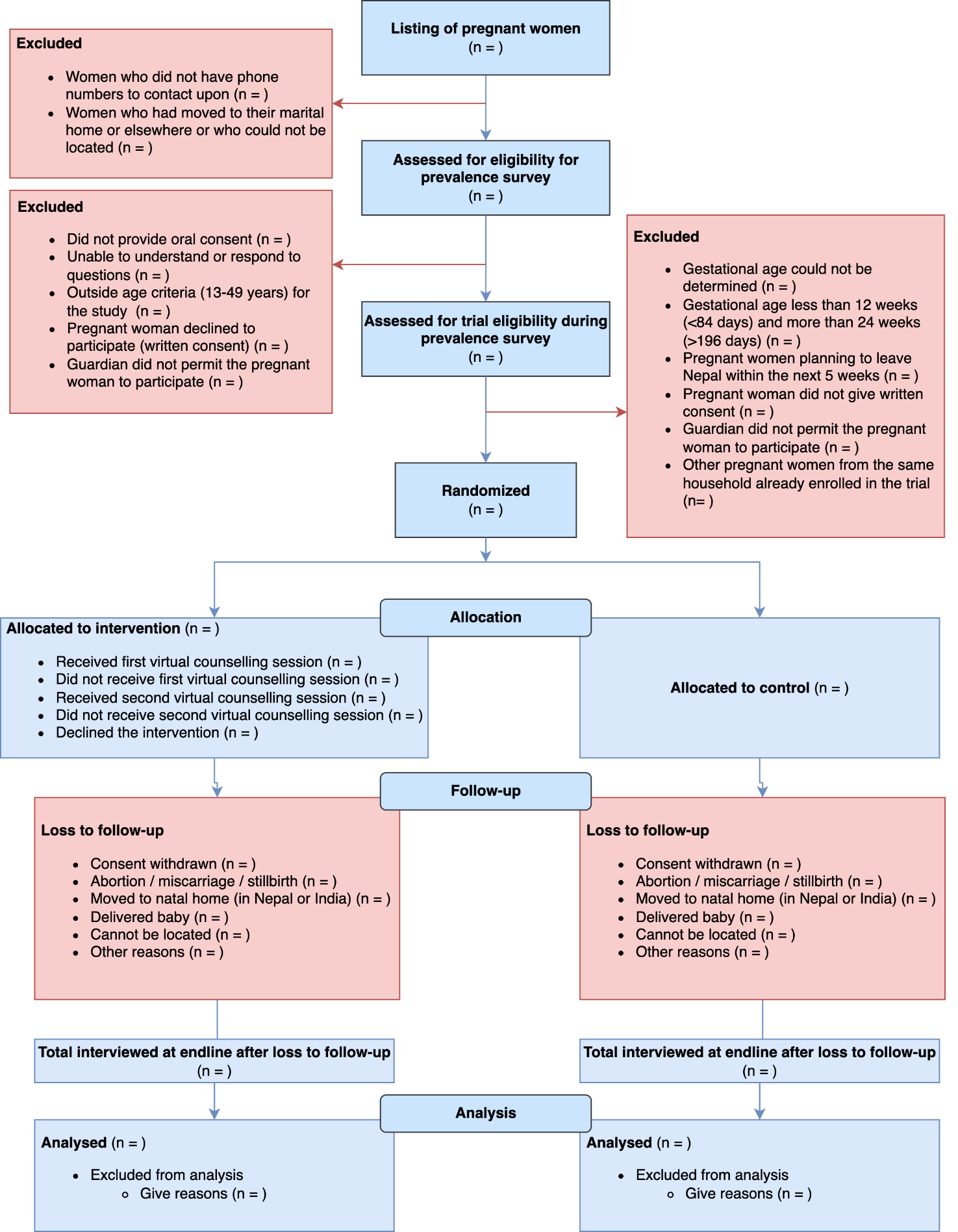


Figure 2. Bar chart of the number of days of IFA consumption in last 14 days by arm at baseline and follow-up by trial arm

**Appendix Table: Associations with loss to follow-up by trial arm**

| **Characteristic** | **Retained in control arm** | | | **Lost to follow-up in control arm** | | | **Retained in Intervention arm** | | | **Lost to follow-up in Intervention arm** | | |
| --- | --- | --- | --- | --- | --- | --- | --- | --- | --- | --- | --- | --- |
|  | **N** | **Freq.** | **%** | **N** | **Freq.** | **%** | **N** | **Freq.** | **%** | **N** | **Freq.** | **%** |
| **Total** |  |  |  |  |  |  |  |  |  |  |  |  |
| **Previous pregnancies** |  |  |  |  |  |  |  |  |  |  |  |  |
| Primigravida |  |  |  |  |  |  |  |  |  |  |  |  |
| 1 previous pregnancy |  |  |  |  |  |  |  |  |  |  |  |  |
| 2 previous pregnancies |  |  |  |  |  |  |  |  |  |  |  |  |
| 3+ previous pregnancies |  |  |  |  |  |  |  |  |  |  |  |  |
| **Pregnant woman’s reading** |  |  |  |  |  |  |  |  |  |  |  |  |
| Cannot read |  |  |  |  |  |  |  |  |  |  |  |  |
| Reads with difficulty or easily |  |  |  |  |  |  |  |  |  |  |  |  |
| **Pregnant woman’s Compliance with IFA** |  |  |  |  |  |  |  |  |  |  |  |  |
| Took recommended iron and folic acid tablet on 12+ out of 14 days at baseline |  |  |  |  |  |  |  |  |  |  |  |  |
| Did not comply |  |  |  |  |  |  |  |  |  |  |  |  |
| **Consumption of intervention-promoted foods** |  |  |  |  |  |  |  |  |  |  |  |  |
| Yes |  |  |  |  |  |  |  |  |  |  |  |  |
| No |  |  |  |  |  |  |  |  |  |  |  |  |
| **Practicing one or more action to enhance bioavailability** |  |  |  |  |  |  |  |  |  |  |  |  |
| Yes |  |  |  |  |  |  |  |  |  |  |  |  |
| No |  |  |  |  |  |  |  |  |  |  |  |  |
| **Pregnant woman’s** | N | Mean | SD | N | Mean | SD | N | Mean | SD | N | Mean | SD |
| Gestational age at enrolment |  |  |  |  |  |  |  |  |  |  |  |  |
| Age (completed years) |  |  |  |  |  |  |  |  |  |  |  |  |
| Dietary diversity  in last 24 hrs (10 groups) |  |  |  |  |  |  |  |  |  |  |  |  |

# References

1. Central Bureau of Statistics N. National Population and Housing Census 2011 (National Report). Kathmandu, Nepal: Government of Nepal, 2012.

2. Ministry of Health Nepal, New ERA, ICF. Nepal Demographic and Health Survey 2016. : Ministry of Health, Nepal, 2017.

3. Wallerstein N, Bernstein E. Empowerment education: Freire's ideas adapted to health education. *Health education quarterly* 1988;15(4):379-94. doi: 10.1177/109019818801500402

4. Moore GF, Audrey S, Barker M, et al. Process evaluation of complex interventions: Medical Research Council guidance. *BMJ* 2015;350:h1258.
