# Supplementary material for: Impact of a virtual antenatal intervention for improved diet and iron intake in Kapilvastu district, Nepal - the VALID randomized controlled trial
Source: Front Nutr. 2024 Nov 7;11:1464967. doi: 10.3389/fnut.2024.1464967 (PMC11580260; doi:10.3389/fnut.2024.1464967)
Supplement: Supplementary file 3 [file Table_3.DOCX]

**Supplementary Annex 3: Associations of baseline characteristics and outcomes with loss to follow-up by trial arm**

| **Outcome or characteristic** | **Control** | | | | | | **Intervention** | | | | | |
| --- | --- | --- | --- | --- | --- | --- | --- | --- | --- | --- | --- | --- |
|  | **Retained** | | | **Lost to follow-up** | | | **Retained** | | | **Lost to follow-up** | | |
|  | N | **Freq** | **%** | N | **Freq** | **%** | N | **Freq** | **%** | N | **Freq** | **%** |
| **Gravida** | 144 |  |  | 17 |  |  | 127 |  |  | 31 |  |  |
| Primigravida | 35 | 30 | 85.7% |  | 5 | 14.3% | 34 | 29 | 85.3% |  | 5 | 14.7% |
| 1 previous pregnancy | 44 | 40 | 90.9% |  | 4 | 9.1% | 44 | 34 | 77.3% |  | 10 | 22.7% |
| 2 previous pregnancies | 39 | 36 | 92.3% |  | 3 | 7.7% | 24 | 16 | 66.7% |  | 8 | 33.3% |
| 3+ previous pregnancies | 43 | 38 | 88.4% |  | 5 | 11.6% | 56 | 48 | 85.7% |  | 8 | 14.3% |
| **Pregnant woman’s reading ability** | 144 |  |  | 17 |  |  | 127 |  |  | 31 |  |  |
| Cannot read | 71 | 62 | 87.3% |  | 9 | 12.7% | 84 | 67 | 79.8% |  | 17 | 20.2% |
| Reads with difficulty or easily | 90 | 82 | 91.1% |  | 8 | 8.9% | 74 | 60 | 81.1% |  | 14 | 18.9% |
| **Pregnant woman’s Compliance with IFA at baseline** | 144 |  |  | 14 |  |  | 127 |  |  | 28 |  |  |
| No | 73 | 63 | 86.3% |  | 10 | 13.7% | 79 | 61 | 77.2% |  | 18 | 22.8% |
| Yes | 85 | 81 | 95.3% |  | 4 | 4.7% | 76 | 66 | 86.8% |  | 10 | 13.2% |
| **Consumption of intervention-promoted foods in last 24h at baseline** | 144 |  |  | 14 |  |  | 127 |  |  | 27 |  |  |
| No | 37 | 36 | 97.3% |  | 1 | 2.7% | 48 | 36 | 75.0% |  | 12 | 25.0% |
| Yes | 121 | 108 | 89.3% |  | 13 | 10.7% | 106 | 91 | 85.8% |  | 15 | 14.2% |
| **Practicing one or more action to enhance bioavailability in last 7d (including tea) at baseline** | 144 |  |  | 14 |  |  | 127 |  |  | 27 |  |  |
| No | 20 | 19 | 95.0% |  | 1 | 5.0% | 19 | 14 | 73.7% |  | 5 | 26.3% |
| Yes | 138 | 125 | 90.6% |  | 13 | 9.4% | 135 | 113 | 83.7% |  | 22 | 16.3% |
| **Continuous variables** | **N** | **Mean** | **SD** | **N** | **Mean** | **SD** | **N** | **Mean** | **SD** | **N** | **Mean** | **SD** |
| Age of woman | 144 | 25.2 | 3.8 | 17 | 25.9 | 5.3 | 127 | 25.6 | 4.6 | 31 | 26.7 | 5.3 |
| Dietary diversity score for women in last 24h | 144 | 4.9 | 1.7 | 14 | 5.7 | 1.3 | 127 | 4.7 | 1.7 | 27 | 4.5 | 1.9 |
| ANC check-ups so far | 144 | 0.9 | 0.8 | 14 | 0.9 | 0.9 | 127 | 0.8 | 0.8 | 27 | 0.8 | 1 |
| **Continuous variables** | **N** | **Median** | **IQR** | **N** | **Median** | **IQR** | **N** | **Median** | **IQR** | **N** | **Median** | **IQR** |
| Gestational age at enrolment | 144 | 18.4 | 15.1, 22.4 | 17 | 18.6 | 13.1,26.3 | 127 | 18 | 15.0, 23.4 | 29 | 16 | 12.9, 22.6 |
